# A Holistic Approach to Enhancing Bakery Products’ Quality and Health Benefits with Saffron Petals—A Review

**DOI:** 10.3390/foods15091521

**Published:** 2026-04-27

**Authors:** Diana-Alexandra Gheorghiu, Liliana Tudoreanu, Liviu Gaceu, Adrian Peticilă, Dana Tăpăloagă, Nicoleta Hădărugă, Adrian Neacșu

**Affiliations:** 1 Faculty of Horticulture, University of Agronomic Sciences and Veterinary Medicine of Bucharest, 59 Marasti Blvd District 1, 011464 Bucharest, Romania; apeticila@yahoo.com; 2 Faculty of Veterinary Medicine, University of Agronomic Sciences and Veterinary Medicine of Bucharest, 59 Marasti Blvd District 1, 011464 Bucharest, Romania; drtapaloaga@yahoo.com; 3 Interdisciplinary Laboratory for Heavy Metals Accumulation in the Food Chain and Modelling, Faculty of Veterinary Medicine, 105 Splaiul Independentei Blvd, District 5, 050097 Bucharest, Romania; 4 Faculty of Food and Turism, Transilvania University of Brasov, 29 Eroilor Blvd, Brasov, 500036 Brasov, Romania; gaceul@yahoo.com; 5 Faculty of Food Engineering, University of Life Sciences “King Mihai I” of Timisoara, 119 Calea Aradului, Timis, 300645 Timisoara, Romania; nico_hadaruga@yahoo.com; 6 Faculty of Midwifery and Nursing, Carol Davila University of Medicine and Pharmacy, 37 Dionisie Lupu, District 2, 050474 Bucharest, Romania; adrianneacsu2006@yahoo.com

**Keywords:** bakery, saffron petals, minerals, bioactives, rheological properties, shelf stability, organoleptic characteristics, therapeutic potentials, analytical methods

## Abstract

As global demand grows for natural health-promoting food ingredients, the agri-food industry’s organic wastes are emerging as promising alternatives thanks to attributes such as biocompatibility, nutritional value and nutraceutical effect. During saffron (*Crocus sativus* L.) production, approximately 53 kg of petals are obtained as a by-product for every 1 kg of saffron spice. The use of saffron petals and petal extracts in bakery products improves products’ shelf life due to the petals’ high content of nutraceuticals and minerals acting as natural preservatives. Petal-enriched bakery products contain high levels of fiber, minerals and antioxidants. Addition of saffron petals into bread dough reduces gluten network strength, increases crumb hardness, enhances acidity, improves water retention, alters color profiles and increases the duration of the shelf life. The formulation for incorporating saffron petals or petal extracts into food products must address three primary criteria: the maximum concentration of bioactive compounds per 100 g of the finished matrix, the thermal stability of these compounds during the baking process, and their bioavailability (in the food matrix) within the human gastrointestinal tract. Nutraceuticals with pharmacological potential are also influenced by the compositional profile: the proximate composition, minerals, phenolic content, flavonols, and antioxidant capacity of saffron petals and bakery products containing saffron petals. Saffron petals exhibit diverse therapeutic potentials, acting as antidepressants, anxiolytics, anticonvulsants, and neuroprotective agents. They also offer metabolic, cardiovascular, hepatoprotective, and renoprotective benefits, along with anti-inflammatory, antimicrobial, and antitumor activities. This article proposes a roadmap for developing bakery products enriched with saffron petals or petal extracts, targeting both pharmacological applications and consumer goods focused on disease prevention and general wellness. This study investigates the biochemical composition of saffron petals and their integration into bakery products. It evaluates the influence of petal-derived additives on rheological properties, shelf stability, and organoleptic characteristics, alongside an assessment of their bioactivity and toxicological profiles. Furthermore, the analytical methodologies employed for ingredient and biological sample characterization are discussed, emphasizing their role in verifying the authenticity, safety, and nutritional functionality of both raw materials and finished formulations.

## 1. Introduction

Saffron (*Crocus sativus* L.) is a perennial geophyte belonging to the Iridaceae family. Saffron (*Crocus sativus* L.) is renowned for its aromatic and therapeutic properties. Saffron is primarily cultivated in the “saffron belt”, stretching from the Mediterranean to Central Asia. Key producers include: Iran (85–90% of global production), India (Jammu and Kashmir, Pampore), with trials in Himachal Pradesh Uttarakhand and Sikkim; ~5% of global production), Afghanistan (Herat Province produces 4% of global production), Greece (Kozani, ~1.5%), Spain (<1%), and Morocco (Taliouine region, ~1%) [1].

Over the last ten years, several European countries have secured PDO (Protected Designation of Origin) certification for their saffron. A PDO certificate guarantees the highest quality, authenticating that all production, processing, and harvesting occurred within a specific region, and used traditional methods. Key European PDO saffron includes Krokos Kozanis (Greece, Western Macedonia), Azafrán de la Mancha (Spain: lbacete, Cuenca, Ciudad Real, and Toledo), Zafferano dell’Aquila (Italy: Piana di Navelli-Abruzzo), Zafferano di Sardegna (Italy), and Zafferano di San Gimignano (Italy: Tuscany). The saffron from Safranbolu Safranı (Turkey) recently attained EU recognition for its high quality and distinction from fraudulent products. Small-scale cultivation is also present in France, Portugal, and, due to climate change, also in Bulgaria, Switzerland, and Romania, with harvesting taking place in autumn. Innovation in Taliouine saffron cultivation is also advancing; a vertical saffron farm is already present in Slovakia [1,2].

POD saffron compositional databases are under development [3], and markers for their authenticity and origin are being researched [4]; however, these efforts are not taking saffron by-products into consideration.

While the spice is derived from the flowers’ stigmas, the remaining floral parts—especially the petals—are typically discarded, having been considered for many years a residue; recent research has pivoted toward their valorization as sources of bioactive compounds and essential minerals [5]. The interest in the utilization of the residues from saffron spice production is driven by the high availability of saffron plant by-products. Producing 1 kg of dried saffron creates a massive amount of organic by-products. Beyond the spice itself, the process generates roughly 63 kg of floral residue (mostly petals, along with stamens and styles) and an additional 1700 kg of field waste, including leaves, spathes, and corm material [6]. Effective waste management has emerged as a pressing global environmental concern, particularly due to the large volumes of organic waste generated by the agri-food processing industry. While saffron stigmas are expensive, the petals are often considered low-value waste. Repurposing them as nutraceuticals and natural preservative creates a “by-product revenue” stream that can account for up to 22% of a saffron farm’s total economic and environmental value.

Recent research has highlighted the nutritional and functional potential of such residues. In this context, the reuse of biomass can contribute to food security, combat malnutrition, and support global sustainability efforts. Studies have demonstrated that saffron petals are rich in carbohydrates, dietary fiber, proteins, and lipids and contain a wide range of antioxidants, phenolic compounds, and essential minerals [7,8,9,10,11].

The increasing demand for natural ingredients and the push toward zero-waste production have stimulated interest in incorporating saffron floral by-products into food formulations, particularly bakery goods. Several studies have demonstrated the successful use of saffron petal extracts in bakery items, yielding products with improved antioxidant activity, color, flavor, and textural properties. These enriched products also show potential for enhancing nutritional profiles and shelf life without compromising sensory quality [9,12].

This article proposes a roadmap for developing bakery products enriched with saffron petals or petal extracts, targeting both pharmacological applications and consumer goods focused on disease prevention and general wellness.

This study investigates the biochemical composition of saffron petals and petal extracts integrated into bakery products. It evaluates the influence of petals and petal-derived additives on the rheological properties, shelf stability, and organoleptic characteristics of bakery products, as well as their bioactivity and toxicological profiles. The investigation of functional bakery systems requires the assessment of analytical consistency as well. Consequently, this study consolidates the main analytical techniques reported in the literature, with particular emphasis on the extraction protocols and characterizations relating to saffron petals, alongside the quality measurement of the resulting bakery products. Advantages and disadvantages of different methods are discussed.

## 2. Methodology of the Review

This study aimed to systematically identify, appraise, and synthesize available research on the compositional characteristics of saffron petals and the quality of bakery products enriched with saffron petals. The review was conducted following the Preferred Reporting Items for Systematic Reviews and Meta-Analyses (PRISMA) statement to ensure transparency, reproducibility, and minimization of bias [13].

The literature search was initially performed in March–May 2024 and subsequently updated in November 2024, November 2025 and January–March 2026; the following databases were used: ScienceDirect, SpringerLink, Web of Science, Scopus, PubMed Central, and Google Scholar. Additional relevant studies were identified through manual screening and citation tracking.

In order to ensure the implementation of the holistic approach proposed, the literature search focused on the following four areas:

(1) Compositional profiles of saffron petals and petal-enriched products;
(2) Impacts of saffron petals on bakery formulations;
(3) Analytical methods for compositional, nutritional, and technological characterization;
(4) Nutritional, functional, and toxicological implications.

Search strings were developed using Boolean operators (AND, OR, NOT) and systematically varied to maximize coverage. The operator NOT was applied where necessary in order to exclude studies focusing exclusively on saffron stigmas, dyes, or unrelated applications.

Below is a list of some of the main keywords, with their combinations included:

- Plant material and extracts: *Crocus sativus* petals; saffron petals; saffron floral by-products; saffron petals extract; *Crocus sativus* petal extract.
- Compositional profile and bioactive compounds: Saffron petals AND phenolic compounds; total phenolic content; flavonoids; anthocyanins; carotenoids; crocin; crocetin; picrocrocin; safranal; antioxidant activity; DPPH; mineral composition; proximate composition; fiber; protein; lipids; ash.
- Processing and extraction: Saffron petals NOT stigmas AND drying; freeze-drying; oven drying; light exposure; UV; storage; extraction; solvent extraction; ultrasound extraction; microwave extraction; supercritical extraction.
- Bakery products and formulations: Saffron petals NOT stigmas AND bread; wheat bread; spelt bread; cookies; biscuits; cakes; bakery products; dough AND formulation; substitution level; fortification; functional food.
- Technological and quality properties: Saffron petals NOT stigmas AND bakery AND texture; texture profile analysis; hardness; cohesiveness; springiness; rheology; dough properties; gluten; water absorption; volume; porosity; color; sensory analysis; hedonic evaluation; consumer acceptance.
- Analytical methods: Saffron petals NOT stigmas AND bakery AND AOAC, AACC; Kjeldahl; Soxhlet; HPLC; GC-MS; spectrophotometry; UV–Vis; ICP-MS.
- Nutritional, functional, and health aspects: Saffron petals AND bakery AND nutrition; functional properties; bioaccessibility; bioavailability; antimicrobial activity; antifungal activity; health benefits; pregnancy; postpartum depression; depression; anxiety; children; elderly; chronic diseases.
- Safety and toxicology: Saffron petals NOT stigmas AND toxic elements; heavy metals; lead; cadmium; mercury; safety; risk assessment.

All keyword combinations were tested in multiple configurations to ensure the retrieval of all relevant studies. This comprehensive strategy allowed the inclusion of studies covering compositional, technological, analytical, and functional aspects of saffron petal incorporation in bakery systems.

All retrieved references were imported into Mendeley for organization, duplicate removal, and screening. Following deduplication, records were screened based on title and abstract, and full-text articles were assessed for eligibility.

- Inclusion criteria: Studies on saffron petals and bakery products enriched with saffron petals; peer-reviewed articles (2010–2026).
- Exclusion criteria: Studies focusing exclusively on saffron stigmas’ utilization in foods and compositional profile; excepted studies also included those focusing on quality assessment, analytical methods; dye applications.

**Figure 1:**
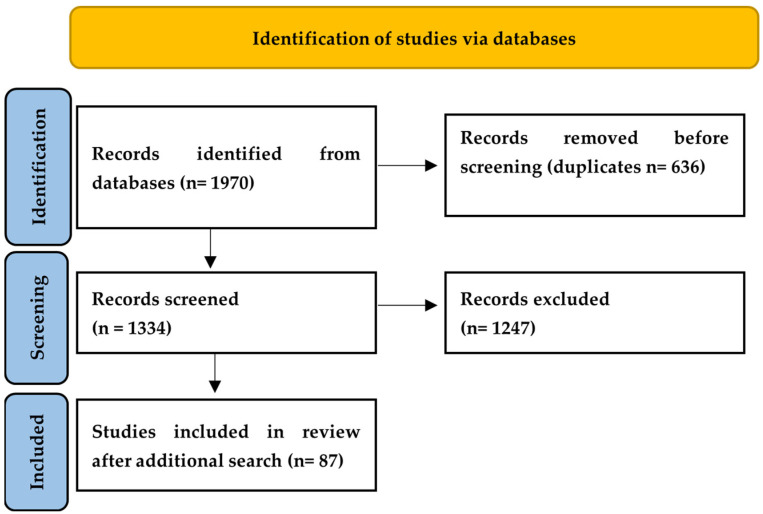
PRISMA (Preferred Reporting Items for Systematic Reviews and Meta-Analyses) flow diagram.

The literature search identified 1970 published articles, from which 87 studies were included after full-text review (Figure 1).

## 3. Saffron Petals and Their Extracts: Composition, Analytical Methods, and Impacts on Bakery Products

### 3.1. Compositional Profile of Saffron Petals, and Analytical Methods

In contrast to saffron stigmas, which are chemically specialized and standardized [14], petals exhibit a more complex and nutritionally relevant composition dominated by flavonoids, anthocyanins, phenolic compounds, and dietary fiber [15,16,17,18,19,20]. However, despite increasing research interest, their compositional characterization remains inconsistent due to variability in the raw material definition, processing conditions, and analytical methodologies [5,21,22].

To highlight the differences between saffron petals and their extracts, and saffron stigmas, Table 1 synthetizes the main compositional features, key bioactive compounds, and biological activities.

#### 3.1.1. Proximate Composition of Saffron Petals, and Analytical Methods

Saffron petals exhibit a nutritionally relevant proximate composition, with carbohydrates ranging from 64.9 to 71.16%, proteins from 6.35 to 8.17%, and dietary fiber between 11.25 and 27.5% [18,27,31]. Ash content remains relatively stable (6.16–7.30%), while lipid content varies between 0.03 and 2.22% [18]. However, these wide ranges indicate strong variability associated with geographical origin, cultivation conditions, and post-harvest processing [32,33,34]. Given the variability in reported proximate composition values, a comparative overview of macronutrient ranges and key compositional parameters is presented in Table 1.

Proximate composition is typically determined using standardized Association of Official Analytical Chemists (AOAC) methods, including oven drying for moisture, Soxhlet extraction for lipids, and incineration for ash [35,36]. While these methods ensure methodological consistency, comparability across studies remains limited due to differences in reporting practices and incomplete characterization, particularly regarding dietary fiber, which is not consistently quantified despite its functional importance.

Crude protein and nitrogen play also important roles in the characterization of saffron petals. Due to the lack of harmonized analytical protocols across studies, a comparative summary of analytical approaches for nitrogen and protein determination is provided in Table 2 to highlight key differences and limitations. In Table 2 is also included information on the methods used for bakery products containing saffron petals or extracts, considering the potential for laboratories to analyze both the petals and the final product.

Protein determination is primarily based on nitrogen quantification using the Kjeldahl method, which remains the reference standard in both plant and bakery product matrices [9,37,38]. However, this method does not distinguish between protein and non-protein nitrogen (NPN), leading to potential overestimation in saffron petals, which contain nitrogenous secondary metabolites.

Alternative techniques, such as Dumas combustion and near-infrared (NIR) spectroscopy, offer advantages in terms of speed and safety. NIR, in particular, is increasingly used for rapid, non-destructive analysis, although it requires calibration against reference methods [40,41,42].

A key limitation in protein determination is the use of a universal nitrogen-to-protein conversion factor (Fp = 6.25), which may overestimate protein content in saffron petals due to the presence of non-protein nitrogen. Lower values (Fp ≈ 5.4) have been proposed for saffron floral residues, highlighting the need for matrix-specific calibration [43].

#### 3.1.2. The Bioactive Profiles of Saffron Petals and Their Extracts, and Analytical Methods

Saffron petals are characterized by a diverse bioactive composition dominated by flavonoids, anthocyanins, and phenolic acids, along with smaller amounts of apocarotenoids. Flavonols and their glycosides, including quercetin, kaempferol, and rutin derivatives, represent the major phenolic class and are largely responsible for the antioxidant activity of petals [16,17,44,45]. Anthocyanins, particularly delphinidin and pelargonidin glycosides, contribute to petal coloration and bioactivity [46,47]. Delphinidin glycosides are the primary pigments in saffron petals, responsible for their characteristic purple, lilac, and bluish hues. These anthocyanins are highly polar and commonly occur as delphinidin 3,5-di-O-glucoside in petal tissues. Pelargonidin glycosides, although present in lower concentrations, contribute to red and orange tonalities and complement the overall color profile. The visible color of these pigments is strongly pH-dependent, appearing red under acidic conditions and shifting toward blue or green in more alkaline environments.

Both delphinidin and pelargonidin derivatives exhibit significant antioxidant activity due to their ability to scavenge free radicals. Delphinidin generally shows higher antioxidant capacity, which is attributed to its greater number of hydroxyl (-OH) groups on the B-ring, which enhance radical stabilization. Glycosylation improves the chemical stability of the petals during processing (e.g., baking) and influences their bioavailability in the human digestive system. In addition to antioxidant effects, these compounds have been associated with anti-inflammatory, anticancer, and neuroprotective activities, with delphinidin reported to modulate signaling pathways involved in oxidative stress and inflammation, as described in Section 4 [46,47].

In addition, simple phenolic acids such as gallic and protocatechuic acids further enhance the functional profile [29,34,48].

Although saffron petals are not a primary source of apocarotenoids, compounds such as crocin, crocetin, picrocrocin, and safranal are detected, albeit at significantly lower concentrations than in stigmas. Their presence is strongly influenced by the processing conditions, particularly drying and extraction, which affect both stability and recovery [30,49].

Metabolomic studies further highlight the compositional complexity of saffron petals, with more than 800 metabolites identified [15]. Compared to saffron stigmas, which are characterized by a terpenoid- and crocin-dominated profile, petals exhibit a composition enriched in flavonoids, anthocyanins, and alkaloids. Notably, certain compounds such as rutin and syringaresinol-di-O-glucoside are specific to petals (Table 3).

Despite the insights provided by metabolomic profiling, direct comparison across studies remains challenging. A major limitation arises from the inconsistent definition of “floral by-products”, with some studies analyzing mixtures of petals, stamens, and styles rather than isolated petals. In addition, differences in extraction techniques, analytical methods, and reporting units further increase the difficulty in the comparison of studies, limiting the establishment of reliable quantitative benchmarks for saffron petal composition [5,21,22].

Quantification of bioactive compounds in saffron petals is predominantly performed using spectrophotometric methods, particularly the Folin–Ciocalteu (F–C) assay for total phenolic content (TPC) and aluminum chloride-based assays for total flavonoid content (TFC) [27,32,45]. These methods are widely applied due to their simplicity, sensitivity, and reproducibility; however, they lack specificity and may overestimate phenolic content due to interference from non-phenolic reducing compounds such as sugars, amino acids, and ascorbic acid [49,50,51].

Considering the variability in analytical protocols, a comparison of the most commonly used methods for the determination of phenolic and flavonoid content is presented in Table 4.

For more precise characterization, advanced analytical techniques such as high-performance liquid chromatography (HPLC), Liquid chromatography–mass spectrometry (LC-MS), and metabolomics approaches have been increasingly applied. These techniques enable compound-specific identification and quantification, revealing detailed flavonoid profiles and confirming the dominance of glycosylated flavonols in saffron petals [15,44,48]. However, their application remains limited compared to spectrophotometric assays, restricting the availability of robust quantitative datasets.

A similar methodological discrepancy is observed in the analysis of carotenoids and related apocarotenoids. While saffron stigma analysis is well-established and relies on advanced chromatographic and spectrometric techniques such as HPLC, Gas chromatography/mass spectrometry (GC/MS), and Ultra performance liquid chromatography tandem mass spectrometry (UPLC-MS/MS) for the accurate quantifications of crocin, picrocrocin, and safranal [30,54,55], these approaches are only rarely applied to saffron petals, mainly in research-oriented laboratories [56].

The methodological differences between saffron petals and stigmas in the analysis of carotenoids and related compounds are highlighted in Table 5.

In contrast, most studies on saffron petals rely on spectrophotometric methods (e.g., UV–Vis at ~450 nm) to estimate total carotenoid content; these methods lack specificity and are prone to interference from co-extracted pigments, particularly anthocyanins. Although advanced techniques such as UPLC-MS/MS provide highly sensitive and selective quantification of individual apocarotenoids, their application to saffron petals remains limited [53]. Emerging extraction technologies, including ultrasound-assisted and supercritical methods, have demonstrated improved recovery of bioactive compounds but are still underexplored as to the petal matrix [7].

The current evidence highlights a methodological gap in petal analysis. While stigma analysis benefits from compound-specific and highly sensitive analytical approaches, saffron petal studies are largely restricted to non-specific spectrophotometric techniques. This limitation hinders accurate compositional characterization and underscores the need for more advanced, standardized, and matrix-specific analytical strategies in future research.

#### 3.1.3. Mineral Profile of Saffron Petals, and Analytical Methods

The mineral composition of saffron petals remains mainly underexplored, as most studies have focused on saffron stigmas or the whole plant matrix. Consequently, detailed multi-element profiles specifically for petals are limited, and the available data are fragmented across studies with differing analytical approaches and aims [5,18,33].

Despite these limitations, saffron petals have been identified as a relevant source of essential minerals, including potassium, calcium, magnesium, and iron, contributing to their nutritional value in both food and feed applications [5,33]. The reported concentrations indicate that petals can provide significant amounts of macro- and microelements while generally remaining within safe limits for toxic elements such as lead and cadmium, as presented in Table 6 [18,62]. However, systematic assessments of toxicological risk specific to saffron petals remain scarce, representing an important gap in the current knowledge.

The influence of agricultural practices on mineral composition remains insufficiently investigated. Limited evidence suggests that environmental factors may play a more dominant role than cultivation system (organic vs. conventional), although dedicated comparative studies are lacking [63]. Furthermore, the long-term effects of climate change and soil degradation on mineral accumulation in saffron petals are largely unexplored, highlighting the need for geographically diverse studies.

In addition to their nutritional role, mineral profiles in saffron petals can serve as a “fingerprint” reflecting soil composition, environmental conditions, and geographical origin [62,64,65]. This has important implications for quality control and authenticity assessment, particularly in the context of high-value products derived from saffron petals.

Mineral concentrations in saffron petals are typically determined using spectrometric and elemental analysis techniques, including inductively coupled plasma mass spectrometry (ICP-MS), atomic absorption spectroscopy (AAS), inductively coupled plasma–optical emission spectrometry (ICP-OES), and, in some cases, neutron activation analysis. The choice of analytical method depends on the required sensitivity, the number of elements to be quantified, and the intended application, such as routine compositional analysis or geographical authentication [66,67]. A comparative overview of the analytical techniques used for mineral determination in saffron petals is presented in Table 7.

Despite the availability of advanced analytical techniques, comparability between studies remains limited due to differences in sample preparation, digestion procedures, and instrumental conditions. In particular, variability between ICP-MS, ICP-OES and AAS measurements, as well as inconsistencies in reporting units, complicate the direct comparison of mineral concentrations across different regions and studies. Therefore, the methodological heterogeneity represents a key limitation in the current literature and underscores the need for harmonized analytical protocols in future research.

#### 3.1.4. Impacts of Sampling, Drying and Extraction Methods on Saffron Petals’ Nutraceutical Quality

The sampling, post-harvest handling, and processing of saffron petals represent critical steps that directly influence both the stability of bioactive compounds and the quality of derived food ingredients. Despite increasing interest in their valorization, these steps remain insufficiently standardized, contributing to variability in the reported compositional data.

Saffron flowers are harvested manually to preserve structural integrity and minimize contamination. However, unlike saffron stigmas—for which processing is well-standardized—handling protocols for petals remain poorly defined. This leads to variability in moisture content, enzymatic degradation, and microbial stability prior to processing [7,21]. Such inconsistencies represent a key limitation affecting reproducibility and cross-study comparability.

Drying is widely recognized as the most critical stabilization step for saffron petals due to their high initial moisture content and rapid post-harvest degradation. Experimental evidence demonstrates that drying conditions strongly affect the retention of anthocyanins and other phenolic compounds. Anthocyanins have been shown to be sensitive to the combination of drying method and time of drying [68].

Traditional air-drying methods have been shown to preserve higher anthocyanin levels, whereas temperatures exceeding 100 °C led to rapid degradation, with significant pigment loss occurring within 10–20 min [68].

More-controlled approaches, including oven drying, vacuum drying, and lyophilization, result in markedly different phytochemical profiles. For example, oven drying at 60 °C allows recovery of minor compounds such as crocins and safranal (<1 mg/g dry weight), while vacuum drying and lyophilization better preserve flavonoids and anthocyanins [39].

Advanced techniques such as electric ovens or autoclaves can reduce drying time to just a few hours. Cerdá-Bernad et. al. (2023) employed a method where freshly harvested flowers were frozen in liquid nitrogen and stored at –80 °C before being dehydrated in a vacuum oven (VACIOTEM, JP SELECTA^®^) at 50 ± 3 °C and 28 mbar for 36 h [18].

As summarized in Table 8, drying methods differ substantially in terms of temperature range, processing time (ranging from several hours to several days), and their impact on compound stability, particularly as to the anthocyanins and flavonoids.

Despite these advances, a major limitation remains the absence of standardized drying protocols. Differences in temperature, duration, and pressure conditions hinder the direct comparison of results and limit industrial reproducibility. Furthermore, few studies evaluate the scalability of optimized drying methods, representing an important research gap [23]. These findings clearly indicate that drying is not only a preservation step but also a selective process that determines the final composition of the petal extracts.

Following stabilization, extraction represents the critical step for recovering bioactive compounds from saffron petals. Conventional techniques such as maceration and Soxhlet extraction remain widely used; however, they are limited by long extraction times and high solvent consumption. In contrast, modern techniques—including ultrasound-assisted extraction (UAE), microwave-assisted extraction (MAE), subcritical water extraction (SWE), and deep eutectic solvent extraction (DESE)—provide improved efficiency, reduced processing time, and enhanced selectivity.

Extraction efficiency and bioactivity are strongly influenced by both the technique and the solvent system [7,30]. Ultrasound-assisted extraction significantly enhances phenolic recovery under optimized conditions (e.g., 96% ethanol, 0.67% citric acid, ~216 W), while reducing extraction time [70]. Similarly, green extraction approaches can recover high levels of flavonoids (up to ~110 mg/g dry plant) and anthocyanins (up to ~16 mg/g), depending on solvent composition and processing parameters.

Solvent polarity plays a key role in determining both extraction yield and the profile of recovered compounds. Aqueous extraction generally provides higher overall yields (up to ~66.3%), but with lower selectivity due to co-extraction of sugars and organic acids. In contrast, hydroalcoholic and ethanolic systems preferentially extract phenolic compounds, resulting in higher levels of antioxidant and antimicrobial activity [16,17,27,31,32]. This is further supported by studies showing that ethanolic extracts exhibit stronger biological activity, including inhibition zones against Gram-positive bacteria [16,27].

Beyond extraction yield and composition, the functional performance of saffron petal extracts in bakery systems is closely linked to the extraction conditions, particularly solvent type and technique, which ultimately influence bioactive availability and technological behavior in the final product. Given the importance of extraction strategy, the effect of solvent type on functional performance is summarized in Table 9.

As detailed in Table 10, extraction techniques differ not only in yield (≈2–49%) but also in selectivity, processing time, solvent requirements, and environmental impact, with green extraction methods offering clear advantages in efficiency and sustainability.

Importantly, extraction conditions also determine compound selectivity. UAE and DESE favor the recovery of flavonoids and anthocyanins, while higher water content promotes co-extraction of sugars and non-antioxidant compounds. This directly affects the functional properties of the extracts, including antioxidant and antimicrobial activity [7,24].

Despite these technological advances, several critical limitations remain. First, extraction protocols are highly heterogeneous, with significant variability in solvent composition, temperature, time, and solid-to-liquid ratio, limiting reproducibility. Second, most studies prioritize extraction yield over functional performance, with limited integration of compositional and biological data. The scalability and industrial feasibility of green extraction techniques remain insufficiently explored, particularly for food applications [25].

#### 3.1.5. Impacts of UV Radiation on the Compositional Profiles of Saffron Petals

From the physiological point of view, saffron plants respond to UV-B radiation by increasing proline and phenolic compounds to shield cellular structures from oxidative damage [71].

Daylight and UV radiation significantly impact the chemical composition of saffron petals and stigmas by altering the concentrations of their primary bioactive compounds, as detailed in Table 11.While moderate exposure can trigger defensive increases in some protective antioxidants, prolonged or intense radiation leads to the degradation of essential quality markers for stigmas and petals, such as crocins and safranal for stigmas, and petals’ anthocyanins and kaempferol derivatives. 

Petals are primarily rich in anthocyanins (purple pigment); specifically, they are rich in delphinidin-3-O-glucoside. While they contain trace amounts of crocins, their primary color degradation from UV-C radiation would involve the breakdown of anthocyanins rather than trigger the “red-pigment” concentration. Petals are more resistant to moderate light than the stigmas; however, they degrade rapidly at temperatures above 100 °C or through intense UV-induced polymerization (browning).

Traditional sun-drying or intense UV exposure causes the breakdown of the purple pigments (anthocyanins) in petals, which are highly sensitive to UV-C and high-heat photo-degradation [71].

The primary drawback of anthocyanins’ presence in the food matrix is their high instability, as they are easily degraded by factors like pH, light, temperature, humidity, and various chemical interactions. These elements can diminish both the concentration and bioactivity of the compounds, ultimately reducing the appeal of the food to consumers [71].

### 3.2. Impacts of the Addition of Saffron Petals and Petal Extracts to Bakery Products, and Analytical Methods

Building on the compositional profile of saffron petals, their incorporation into bakery products allows the transfer of bioactive compounds and nutrients into food systems, although their stability and functionality are influenced by processing conditions.

The incorporation of saffron petals into bakery products represents a promising approach for the valorization of floral by-products, contributing to the development of functional and clean-label foods.

A very large number of studies have reported the use of saffron stigmas, extracts and encapsulated bioactives from stigmas for the development of bakery products. Despite the increasing interest in saffron-derived ingredients, the number of studies specifically investigating saffron petals in bakery systems remains very limited, with available evidence largely restricted to bread formulations [9,12].

Table 12 presents a global overview of the compositional, functional, and technological effects of saffron petal incorporation in bakery systems.

#### 3.2.1. Proximate Composition of Bakery Products Containing Saffron Petals, and Analytical Methods

The incorporation of saffron petals into bakery products results in consistent but moderate modifications of proximate composition (Table 12). The most pronounced effect is the increase in dietary fiber, with reported improvements of approximately 25–30%, while protein, ash, and moisture contents show relatively minor variations depending on formulation and substitution level [9].

Carbohydrates remain the dominant fraction (~60–70%), reflecting the cereal-based matrix, whereas lipid content is largely determined by formulation rather than petal addition. Compared to raw petals, these values reflect dilution effects and the predominance of cereal-derived components [12].

As discussed for saffron petals, the presence of non-protein nitrogen compounds may lead to overestimation of protein when using a standard conversion factor (Fp = 6.25), which should be considered when interpreting bakery product data [43].

Proximate composition is typically determined by using standardized AOAC methods, ensuring methodological consistency across studies. Moisture is measured by oven drying (105 ± 5 °C), ash by incineration (550 ± 25 °C), lipids by Soxhlet extraction, and protein by the Kjeldahl method using a conversion factor of 6.25. Carbohydrates are calculated by difference, while dietary fiber is determined using enzymatic–gravimetric methods, when reported [9,12]. Table 13 summarizes these standardized procedures.

While AOAC-based methods provide a robust analytical framework, they do not capture structural modifications occurring in bakery product matrices. Carbohydrates calculated by difference accumulate analytical variability, and differences in moisture determination protocols may introduce deviations, particularly in fiber-rich systems [9,12]. In addition, dietary fiber is not consistently reported, limiting cross-study comparability.

From a formulation perspective, increasing petal incorporation from 2.5% to 10% enhances nutritional value, with dietary fiber increases of up to 30% and improvements in mineral content, including potassium (~162 to 277–289 mg/100 g) and iron (~2 to 15–18 mg/100 g) [9]. These improvements are accompanied by physicochemical changes such as increased acidity (~0.12% to 0.28%) and reduced pH (~5.5 to 5.2), indicating a trade-off between nutritional enhancement and technological performance.

Whole petal incorporation primarily affects structural and nutritional properties, whereas extract-based systems enhance functional attributes without significantly altering macronutrient composition. Encapsulation strategies may further improve stability of bioactives, but introduce additional formulation complexity. A comparative overview of these incorporation strategies is presented in Table 14.

#### 3.2.2. Bioactive Compounds in Bakery Products Containing Saffron Petals and Analytical Methods

The incorporation of saffron petals into bakery products leads to a consistent enhancement of bioactive compounds, particularly phenolics and flavonoids (Table 12). Total phenolic content (TPC) typically reaches approximately 1.2–2.4 mg GAE/g dry weight, corresponding to up to a twofold increase compared to control formulations [9]. However, quantitative data remain limited, and results are often expressed as relative increases rather than absolute concentrations, restricting direct comparison across studies.

Given that saffron petals are rich in flavonoids and anthocyanins, the observed increase in antioxidant capacity in bakery products is likely associated with the partial retention of these compounds, although compound-specific data are not available.

In bakery systems, the analysis of bioactive compounds relies predominantly on spectrophotometric methods, including the Folin–Ciocalteu assay for total phenolic content and antioxidant assays such as DPPH, ABTS, and FRAP. These methods are widely applied due to their simplicity and reproducibility but lack specificity and are strongly influenced by matrix effects, including the presence of sugars, proteins, and Maillard-reaction products [49].

In contrast, advanced analytical techniques such as HPLC and LC-MS are primarily applied to raw saffron petals for compound-specific characterization [15,16,17,44] and remain largely underutilized in bakery product matrices.

Table 15 summarizes the analytical methods applied in saffron petal-enriched bakery products, based on the studies of Cerdá-Bernad and Frutos (2023) and Ziaee Rizi et al. (2024), together with commonly used analytical approaches in other bakery systems, which are included for the purposes of comparison [9,12].

Although widely used due to their simplicity and reproducibility, these methods lack specificity and are strongly affected by matrix effects inherent to complex bakery systems, which may lead to overestimation of phenolic content. In addition, thermal processing during baking influences both the stability and extractability of phenolic compounds, contributing to variability in reported values [9,12].

Ethanolic and hydroalcoholic extracts generally exhibit stronger biological activity, including lower IC_50_ values and enhanced antimicrobial effects, compared to aqueous extracts. This is attributed to their higher efficiency in extracting phenolic compounds such as flavonoids [16,17,37]. These differences are particularly relevant for bakery applications, in which extract composition directly influences oxidative stability, microbial inhibition, and product shelf life. However, solvent selection must also consider food safety, regulatory constraints, and potential impacts on product formulation.

The data presented in Table 14 demonstrate that extraction strategy is a critical factor in optimizing the functional performance of saffron petal-derived ingredients. While aqueous systems maximize extraction yield, ethanolic and hydroalcoholic systems provide higher concentrations of bioactive compounds and enhanced biological activity, making them more suitable for applications targeting antioxidant and antimicrobial functionalities [7,24,30].

The use of extracts reflects the extraction strategies described for saffron petals, in which solvent and technique significantly influence the recovery of bioactive compounds and their functional performance in bakery systems. Nevertheless, variability in extraction conditions, solvent composition, and reporting units across studies limits direct comparison of results, highlighting the need for standardized extraction protocols and analytical approaches [24,54,72].

In addition to extract-based systems, whole-petal incorporation contributes both structural and functional effects, while encapsulation approaches may further improve the stability of bioactive compounds during processing and storage [21]. However, these strategies remain underexplored in bakery product matrices and require further investigation.

This contrasts with saffron petal characterization studies, in which advanced chromatographic and metabolomic techniques are widely applied [15], highlighting a methodological gap between raw material analysis and finished bakery products.

#### 3.2.3. Mineral Composition of Bakery Products Containing Saffron Petals, and Analytical Methods

Beyond macronutrient composition, saffron petals contribute micronutrients that enhance the nutritional profiles of bakery products.

The incorporation of saffron petals results in a measurable increase in mineral content. In bread systems, potassium levels range from ~270 to 290 mg/100 g, calcium from ~90 to 95 mg/100 g, magnesium from ~40 to 50 mg/100 g, and iron from ~15 to 18 mg/100 g [9]. Table 16 highlights these mineral contributions to bakery products.

Variability across studies is influenced by formulation parameters and incorporation levels. Trace elements such as Zn, Cu, and Mn are not reported, while data on toxic elements (As, Pb, Cd, Hg) are generally lacking with reference to bakery systems. However, evidence from raw petal analyses indicates that these elements are present at trace levels within safety limits, suggesting no significant risk upon incorporation [73].

Analytical determination is typically performed using spectrometric techniques such as AAS or ICP-based methods, following acid digestion. However, differences in digestion protocols and instrumentation introduce variability, limiting comparability across studies [66,67].

Unlike saffron petal analysis, in which advanced techniques such as ICP-MS are used for detailed elemental profiling, mineral analysis in bakery products is limited to basic compositional determination. This reflects a broader gap in the literature, where mineral composition is less systematically evaluated than bioactive compounds in saffron petal-enriched bakery systems.

#### 3.2.4. Color, Sensory and Textural Properties of Enriched Bakery Products, and Analytical Methods

Color is one of the most immediate and influential quality attributes in saffron petal-enriched bakery products, directly affecting consumer perception and acceptance. Instrumental color evaluation is typically performed using the CIE L*, a*, b* system, which quantifies lightness (L*), redness/greenness (a*), and yellowness/blueness (b*).

Across studies, color measurements are conducted using different instruments, including spectrophotocolorimeters, Chroma Meters, and Hunter Lab colorimeters, although all rely on the same CIELAB color space. While these approaches are well-established in bakery research, their application in saffron petal-enriched systems is primarily documented in bread formulations [9].

The incorporation of saffron petals consistently results in decreased L* values and increased a* and b* values, indicating darker products with enhanced red–yellow pigmentation. These changes are attributed to the presence of anthocyanins, flavonoids, and other phenolic compounds, as well as to Maillard reactions during baking. The magnitude of these effects is strongly dependent on incorporation level and formulation strategy.

Textural properties are primarily evaluated using texture profile analysis (TPA), often complemented by porosity and volume measurements. Table 17 presents the analytical approaches used for textural characterization of saffron petal-enriched bakery products.

The available evidence indicates increased hardness and reduced elasticity with higher levels of petal incorporation, reflecting disruption of the gluten–starch network by fiber and phenolic compounds [9]. In extract-based systems, hardness may increase substantially, whereas fermentation strategies can mitigate these effects, reducing hardness to approximately 10.21 N and improving crumb porosity to 16.16% [12].

These structural modifications are associated with interactions between petal-derived fiber and gluten proteins, which weaken the viscoelastic network and reduce gas retention. As a result, saffron petal-enriched bakery products exhibit greater variability in textural properties, compared to conventional systems.

Despite the widespread use of TPA, methodological variability remains a significant limitation. Differences in probe type, compression parameters, and sample preparation reduce reproducibility and limit cross-study comparability. In addition, correlations between instrumental measurements and sensory perception are not systematically established, restricting the interpretation of technological relevance.

Sensory evaluation is typically conducted using hedonic and descriptive tests, assessing attributes such as color, aroma, taste, texture, and overall acceptability. Table 18 summarizes sensory evaluation methods and key findings in saffron petal-enriched bakery products.

Across studies, optimal consumer acceptance is consistently reported at 2.5–5% petal incorporation, while higher levels (≥10%) lead to reduced acceptability due to increased bitterness, firmer texture, and more intense coloration [9,12]. Extract-based formulations may enhance flavor complexity but can also introduce variability, depending on concentration and extraction method.

Compared to conventional bakery products, sensory analysis in saffron petal-enriched systems is less standardized, often relying on small, semi-trained panels and variable evaluation conditions. This limits reproducibility and comparability across studies.

A key limitation across all quality attributes is the lack of integration between instrumental and sensory data. While instrumental methods provide objective measurements, they are not consistently correlated with consumer perception, which remains essential for product acceptance.

While conventional analytical methods for color, texture, and sensory evaluation are applicable to saffron petal-enriched bakery products, their implementation lacks standardization. This highlights the need for harmonized methodologies and integrated instrumental–sensory approaches to enable reliable quality assessment and support product development.

#### 3.2.5. Multicriteria Decision Analysis (MCDA)

Multicriteria Decision Analysis (MCDA), also referred to as Multiple-Criteria Decision-Making (MCDM), is not a singular method, but rather a comprehensive conceptual framework encompassing various tools designed to support complex decision-making processes involving multiple, often conflicting, criteria [74]. Although rooted in computer science and decision theory, MCDA has been widely adopted across diverse domains, including food technology, where it facilitates structured evaluation of alternatives based on economic, environmental, and functional considerations [74]. In one such application, MCDM was utilized to assess the suitability of food industry by-products—specifically grape pomace, apple pulp, and defatted hemp seed meal—as raw materials in bakery products. These alternatives were evaluated based on six criteria: regional availability, market price, nutritional value, thermal and processing stability, sensory compatibility, and impact on product shelf life, enabling a transparent and evidence-based selection process [75]. A more advanced methodological integration was demonstrated by Serpa et al. (2022), who applied a hybrid Fuzzy AHP–Fuzzy TOPSIS framework to guide sustainable decision-making in a medium-sized bakery [76]. Their empirical study involved defining four sustainability-oriented criteria—quality, cost, production time, and resource use—selected through literature review and expert consultation. Data were collected via structured questionnaires from six experienced decision-makers, and the hybrid fuzzy methods were employed to handle uncertainty in judgment while enabling a nuanced ranking of sustainability-driven alternatives [76].

By applying MCDA frameworks in bakery science, researchers intend to find the “Goldilocks zone” of fortification. The “Goldilocks” principle in food science refers to a “just right” approach in which a process, ingredient concentration, or condition is optimized to be neither too high nor too low, ensuring maximum quality, safety, or efficiency. In bakery science, the Goldilocks principle applies to achieving the perfect balance in texture, flavor, and shelf-stability. This approach is grounded in the fact that increasing petal concentration boosts health benefits, but eventually degrades crumb elasticity and flavor. Using statistical software [77,78] enables researchers to find the exact mathematical intersection where antioxidant activity is maximized while sensory score and specific volume remain above acceptable commercial thresholds.

This integrated approach showcases the methodological flexibility and practical utility of MCDM techniques in enhancing decision quality, particularly in contexts that balance sustainability, resource optimization, and production efficiency.

## 4. Beneficial and Toxicological Effects of Saffron Petals and Petal Extracts

The use of saffron petals as an addition to food products is known to improve the nutritional quality and shelf life of these products. In the last five years, several studies have shown their pharmacological benefits, such as immune stimulation, hypolipidemic qualities, antitumor activities, and antiviral symptoms in cases of severe COVID-19 infection [5].

The flavonoid kaempferol is a major constituent of saffron petals (*Crocus sativus* L.), and exhibits significant antihypertensive activity through several distinct mechanisms. Research indicates that these petals are a cost-effective source of this bioactive compound, which works to lower blood pressure and protect the cardiovascular system.

There is limited information on the therapeutic effects of saffron petals, as such, within the clinical contexts of pregnancy, the puerperium, and the physiological sequelae of human senescence, which are factors characteristic of two vulnerable populations: pregnant women and the geriatric demographic. The use of saffron petals could be a solution for type 2 diabetes mellitus in pregnancy, the prevalence of which is increasing, mainly because of the rise in maternal obesity, with pregestational diabetes occurring in one to two percent of all pregnancies.

Animal toxicological studies indicate that while food-level consumption of saffron petals is generally safe, high doses may exert uterotonic effects or effects on the reproductivefunctions, supporting recommendations against supplemental use during pregnancy. Comparative studies on saffron petals demonstrate lower toxicity, showing differences in phytochemical potency among plant parts. Together, these findings suggest that while culinary use of stigmas is safe, supplementation during pregnancy or lactation should be approached with caution until further human safety data are available. However, the use of saffron petals represents a lower toxicological risk [10].

Generally speaking, the nutraceutical effect of saffron petals may cover a large number of therapeutic areas such as depression, diabetes, blood pressure, anti-arthritis, cholesterol control, and digestion. These conditions are among the most commonly diagnosed in the elderly, as well as during pregnancy and the postpartum period [79,80].

Although there are reports on saffron petals’ pharmacological effects, studies focusing on the bioavailability of the minerals in saffron petals when used in human or animal diets are virtually nonexistent. Mineral deficiencies can affect neurotransmission, inflammation, oxidative stress, and hormonal systems that are implicated in postpartum depression, so mechanistic links are biologically plausible and supported by observational data. To date, no information is available on the combined effects of minerals and nutraceuticals from enriched bakery products with saffron petals or the extract of petals for humans. These studies are essential to validate the nutritional and therapeutic efficacy of the minerals in saffron petals, this being important especially for vulnerable groups (the elderly, or those in pregnancy or postpartum pathology).

*Crocus sativus* L. petals exhibit various pharmacological effects, which are described in Table 19.

Toxicological studies in mice report that the intraperitoneal LD_50_ (lethal dose for 50% of test subjects) for saffron stigma and petals are approximately 1.6 g/kg and 6 g/kg, respectively [18]. However, when administered orally, saffron petals exhibit low toxicity, with an LD_50_ greater than 5 g/kg.

In humans, ingesting less than 1.5 g of saffron petals is generally considered non-toxic. Toxic effects typically emerge at doses exceeding 5 g, while ingestion of around 20 g per day can be fatal. For petal extracts, mild toxicity may result in symptoms such as dizziness, nausea, vomiting, and diarrhea. In more severe cases, the toxicity of the extract of the petals can manifest as numbness, tingling in the extremities, and yellowing of the skin and eyes due to deposition of the saffron’s yellow pigments on the skin and conjunctiva [12]. Polyphenols exist in large quantities in saffron petals; while beneficial in diet, high-dose phenol products can be harmful, as some people may have difficulty processing phenols due to phenol sulfotransferase (PST) deficiency.

Animal studies have demonstrated that very high doses of saffron stigma or its volatile constituents may induce adverse effects, including changes in reproductive hormone levels and uterine smooth muscle stimulation, supporting traditional cautions against excessive intake during pregnancy [26,86,87]. These findings underpin current recommendations to avoid medicinal or supplemental doses of saffron stigmas during gestation, particularly in the first trimester, while acknowledging that culinary-level exposure is substantially lower than doses associated with toxicity in experimental models.

Clinical trials have demonstrated that 30 mg/day of petal extract is as effective as fluoxetine for mild-to-moderate depression, likely due to its modulation of the serotonergic system. Petals contribute to lowering blood pressure by reducing peripheral resistance and exhibit antidiabetic properties by lowering fasting blood sugar and blood urea nitrogen [47]. However, a great majority of the reported data are from animal or in vitro studies.

Prenatal and developmental toxicity studies conducted in rodents further clarify the safety margins of saffron exposure. High-dose oral administration of saffron preparations during organogenesis in rats did not consistently result in maternal toxicity, fetal malformations, or growth retardation, suggesting a relatively wide therapeutic window under controlled conditions [85,86]. However, extrapolation to human pregnancy remains limited by interspecies differences, extract composition, and dosing regimens that exceed typical dietary intake. As a conclusion: stigma extracts have higher toxicity, when compared to petal extracts. Consequently, while these animal data do not indicate strong teratogenic risk, they reinforce a precautionary approach to the use of saffron petals as functional ingredients or supplements during pregnancy [85,86]. To date there is only very limited information on the toxicity of extracts or powders of petals during pregnancy or postpartum.

Comparative toxicological studies on saffron petals provide additional context for petals’ safety. Petal extracts have demonstrated lower acute and subacute toxicity profiles in animal models, with high LD_50_ values and minimal hepatic, renal, or hematological alterations, even at elevated doses [26].

Although petals differ phytochemically from stigmas, their favorable toxicological profile supports the broader conclusion that saffron bioactives exhibit relatively low systemic toxicity when administered within reasonable limits [20]. Conversely, the higher concentration of bioactive compounds in the stigmas necessitates increased clinical vigilance when such products are administered to pregnant or postpartum patients [20,85,86].

Although, for the postpartum period, clinical trials using standardized saffron stigma extracts have shown efficacy in reducing symptoms of mild-to-moderate postpartum depression, with tolerability comparable to conventional antidepressants [85,86], no studies have been found that identify the effects of the addition of petals to bakery products. No serious adverse effects in mothers have been reported; however, data on infant exposure via breastfeeding remain insufficient. Therefore, while petal-derived preparations may represent promising food-derived nutraceuticals or pharmacological products for postpartum mental health, their use should be guided by dose control and clinical supervision until more comprehensive lactation safety data become available.

## 5. Conclusions

Lately, increasing attention is being directed toward underutilized saffron floral parts—such as petals and stamens.

Although POD saffron stigmas are known and markers for their authenticity and origin have been identified, compositional databases and markers for the respective floral residues are lacking. Their integration into bakery products takes into consideration also the origin of the saffron ingredients. There is clear evidence on the existence of differences in bioactive compounds between saffron petals of different origins.

The integration of saffron floral by-products into food systems represents a promising and sustainable strategy for enhancing the nutritional and functional profiles of food products.

Saffron petals are rich in flavonols and glycosides, anthocyanins, phenolic acids and related phenolics, with low concentrations of apocarotenoids and several monoterpenoids, as well as unique petal metabolites such as quassin, rutin and syringaresinol-di-O-glucoside having been identified by isolation studies and metabolomics.

Information on the total mineral concentrations in saffron petals is limited. Most studies refer to macronutrients such as potassium, calcium, and phosphorus and essential trace elements such as iron, zinc, and magnesium. Available evidence indicates that potentially toxic elements, cadmium, lead, mercury, arsenic, and chromium, have been found to be within regulatory limits.

Valorizing saffron petals in bakery requires a comprehensive analytical approach. As standard spectrophotometric tests like the Folin–Ciocalteu method may overestimate total phenolic content, more advanced techniques and methods (such as HPLC-MS/MS) are needed for accurate quantifications of phenolic compounds, anthocyanins, and apocarotenoids, as well as pH-differential methods for pigments, and antioxidant assays like ORAC and FRAP can be used to track compound stability through thermal processing [56]. Advanced methods such as NMR (nuclear magnetic resonance) and DNA-based approaches protect the product’s integrity by detecting potential adulteration with cheaper flowers like safflower or marigold. Although the ISO 3632 standard provides a baseline for identifying the quality and purity of saffron, to date, there is no standardized method for the evaluation of the quality of saffron petals.

While drying and extraction technologies for saffron petals have advanced considerably, the lack of harmonized and scalable methodologies remains a major bottleneck. Future research should focus on optimizing process parameters in a holistic manner, linking processing conditions to compositional outcomes and functional performance to ensure the consistent quality and applicability of saffron petal-derived ingredients in food systems.

Recent studies have demonstrated that the incorporation of saffron petals up to 5% into bakery products, particularly wheat- and spelt-based breads, can significantly improve antioxidant capacity, dietary fiber content, and the overall nutritional value and shelf-life, without adversely affecting sensory attributes. The adverse effects can be identified starting at a 10% addition.

While a considerable number of studies have focused on the use of saffron stigmas, their extracts, and encapsulated bioactive compounds in bakery products, research on saffron petals remains limited. To date, only a very small number of studies have specifically investigated the use of saffron petals in bakery products, revealing a substantial gap in the literature and a clear opportunity for further exploration.

Saffron petals may exhibit pharmacological effects aimed at disease intervention, treatment, or curing a specific pathology, as well as nutraceutical effects focused on prevention, health maintenance, and reducing the risk of disease.

Several main therapeutic pathways have been identified specifically for petal extracts, such as antidepressant efficacy (30 mg/day of petal extract is as effective as fluoxetine for mild-to-moderate depression, likely due to its modulation of the serotonergic system); antispasmodic and myorelaxant effects (petal extracts have shown significant inhibition of smooth muscle contractions by blocking muscarinic and adrenergic receptors); anti-inflammatory and antinociceptive effects (high levels of flavonoids and tannins reduce chronic inflammation and chemical-induced pain in animal models); and metabolic and cardioprotective effects (petals contribute to lowering blood pressure by reducing peripheral resistance and exhibit antidiabetic properties by lowering fasting blood sugar and blood urea nitrogen).

Despite these properties, clinical data for specific demographics remain sparse. A very low number of studies report the use of saffron petals as being associated with positive health effects in the postpartum period and in pregnant women. While stigma extracts show promise for postpartum depression, data on petals for this group is limited. The neuroprotective and antioxidant effects of petal-derived kaempferol suggest the potential for treatments for age-related disorders, yet human trials are significantly lacking.

Evidence regarding the clinical utility of saffron petals remains sparse, particularly concerning postpartum outcomes. Furthermore, pregnant, postpartum, and geriatric populations are critically underrepresented in the current literature, highlighting a significant gap in the available data

There is very limited information on the investigation of the pharmacological effects of saffron petals in humans; most studies focus on the nutraceutical effects. Very little information was found on the use of saffron petals as a supportive therapy, alongside conventional medicine, used to reduce drug dosages and side effects.

Moreover, there is very little information on the synergic effects of minerals and bioactives in the study of the pharmacological or nutraceutical effects of the addition of saffron petals into bakery products.

## 6. Future Research Priorities

Future research priorities should address the critical gaps between laboratory findings and commercial viability.

Based on the current scientific evidence, several research areas have been identified which need to be addressed to advance the use of saffron floral by-products in the bakery industry for the development of products with nutraceutical effect.

One important research area refers to thermal stability and processing optimization, attempting to understand what the specific degradation kinetics of saffron petal pigments and phenols are at standard baking temperatures (180–220 °C), as it has been demonstrated that anthocyanins are unstable at temperatures above 100 °C. More research is needed to study encapsulation or pre-treatment techniques used to protect these sensitive molecules during the baking process without compromising bread texture.

The synergy with other “super-ingredients” needs special attention. Research should explore a larger number of minerals (including trace minerals with important roles in human metabolism) in saffron petals and bakery products containing saffron petals. Furthermore, there is a lack of data to confirm that the synergistic effect of combining petals with other natural antioxidants yields a more bioactive functional food, as compared to petals as a monotherapy. The synergistic health or quality effects when saffron petals are combined with other functional ingredients need more research, as this area has been rarely addressed.

Future research also has to address bioaccessibility and matrix interaction, explaining how the complex structure of different bakery product matrices influences the bioaccessibility of bioactive substances such as kaempferol and anthocyanins. The health benefits of antioxidants from saffron petal extracts or from saffron petal powder depend on the availability of bioactive substances through the digestive tract. For example, research must determine if the fermentation metabolites in sourdough enhance or hinder the release of these bioactive compounds during human digestion.

Advanced characterization of raw material has to be used. Using LC-MS/MS (liquid chromatography–mass spectrometry) for fingerprinting or LC-MS/MS allows for the exact identification of specific kaempferol glycosides and crocetin derivatives unique to the petals, ensuring batch-to-batch consistency for industrial standardization.

The most-used sensory methods used by panels examining bakery products enriched with saffron petal are: Quantitative Descriptive Analysis (QDA), Flash Profile, and Simple Descriptive Test. Saffron petals introduce complex floral and bitter notes, so therefore Temporal Check-All-That-Apply (TCATA) or Progressive Profiling should be used instead, as they can track how those flavors evolve while the consumer is chewing and how they change as the bread grows stale over 7 days. More advanced methods such as Dynamic Sensory Profiling are underused too.

The strategic roadmap presented (Appendix A) in this study serves as a bridge between existing research and the future of functional bakery products. By taking into consideration the research gaps identified, this framework provides a clear, actionable pathway toward high-quality, sustainable bakery products. This approach supports the enhancement of bakery product value and also promotes the efficient use of saffron by-products in a circular economy.

## Figures and Tables

**Table 1:** Comparative compositional profile of saffron petals, saffron petals’ extracts and saffron stigmas.

Component	Saffron Petals	Saffron Petals Extract	Saffron Stigmas	Reference
Main Bioactives	Flavonoids 60.64 CE/g; Phenolics 65.34–677.7 mg GAE/g	Phenolics 3.09 mg GAE/g Flavonoids 0.92 mg QE/g IC_50_ 235 µg/mL	Crocin (major) Phenolics 8.28–35.69 mg GAE/100 g Flavonoids 3.53–14.10	[7,16,17,23,24]
Key Markers	Metabolites: flavonoids, anthocyanins	Antibacterial 10.66–22 mm inhibition zone MIC for bacteria 4.33–5.62 mg/mL 2(5H)-Furanone 92.10% Safranal 3.56% Limonene 1.48%	Crocin/picrocrocin/safranal markers	[16,17,25,26]
Minerals/Nutrients	Protein 6.35–8.17 g/100 g; Carbohydrates 64.9–71.16 g/100 g; Lipids 0.03–2.22 g/100 g; Ash 6.16–7.30 g/100 g Fiber 11.25–27.5 g/100 g Na 45.85–120 mg/kg K 23.75–13,530 mg/kg; Ca 39.25–1250 mg/kg; Fe 149.5–280 mg/kg Zn 47.23 mg/kg	Not quantified	Protein 5.97–13.63 g/100 g Carbohydrates 62 g/100 g Lipids 0.03–8.76 g/100 g Ash 6.6–13.45 g/100 g Fiber 13.38–13.8 g/100 g Na 53.35–100 mg/kg K 26.35–14,860 mg/kg Ca 14.8–1070 mg/kg P 3270 mg/kg Fe 94.76–110 mg/kg Zn 49.96 mg/kg	[7,18,26,27]
Biological Activity	Antioxidant Antibacterial (moderate)	Strong antioxidant (IC_50_ 235 µg/mL) Antimicrobial (up to 22 mm inhibition) Antifungal; anticancer; anti-tyrosinase	Antioxidant; antidepressant; neuroprotective; anti-inflammatory; anti-ulcer; anticancer	[16,17,26,28]
Processing/Extraction Influence	Strong variability depending on drying method, origin, and particle size	Solvent-dependent extraction yield and composition; hydroalcoholic extracts show higher phenolic recovery	Standardized drying and processing conditions	[7,24,26,29,30]
Standardization	Not standardized	Not standardized	ISO 3632 (crocin, picrocrocin, safranal)	[14]

**Table 2:** Comparison between Kjeldahl, Dumas and NIR methods for nitrogen determination in petal samples and bakery products.

Method	Principle	Time	Accuracy	Main Advantages	Main Limitations	Relevance	References
Kjeldahl	Wet digestion and titration	1–3 h	High	Standard reference method	Time-consuming; hazardous reagents; may overestimate protein (NPN)	Most widely used	[9,37,38]
Dumas	Combustion and gas detection	4–5 min	High	Rapid; no hazardous chemicals	Expensive equipment	Good alternative	[39]
NIR	Spectroscopic prediction (calibrated)	Seconds	Moderate	Fast; non-destructive	Requires calibration; lower accuracy in complex matrices	Suitable for screening	[40,41,42]

**Table 3:** Major bioactive compounds in Chinese Saffron (*Crocus sativus* L.) petals and stigmas, based on the data published by Zhou et al. (2022) [15].

Feature	Petals	Stigmas
Total Metabolites	824	827
Shared Metabolites	819	819
Unique Metabolites	Quassin Rutin Syringaresinol-di-O-glucoside	(+)-affinisine Xanthyletin Nervonic acid Vitexicarpin Poncirin Pseudouridine Hygromycin B Leucodin
Primary Profile	Rich in flavonoids and alkaloids	Rich in terpenoids and crocins

**Table 4:** Comparative analytical methods for the determination of bioactive compounds in saffron petals and extracts.

Parameter	Method	Principle	Key Conditions	Output Expression	Main Limitations	Reference
Total phenolic content (TPC)	Folin–Ciocalteu (F–C)	Redox reaction with phenolics	725–760 nm; Na_2_CO_3_; methanol/PBS extracts	mg GAE/g	Non-specific; reacts with reducing compounds (ascorbic acid, amino acids)	[9,27,52]
Total flavonoid content (TFC)	AlCl_3_ colorimetric assay	Complex formation with flavonoids	415–510 nm; AlCl_3_; incubation 30 min	mg QE/g	Low specificity; interference from co-extracted compounds	[11,29,32,34]
Total carotenoid content (TCC)	UV–Vis spectrophotometry	Light absorption by carotenoids	~450 nm; acetone/petroleum ether extraction	µg/g or mg/g	Cannot distinguish individual carotenoids; pigment interference	[29,30,34]
Individual phenolics/flavonoids	HPLC-PDA/HPLC-ESI-MS	Chromatographic separation + spectral detection	Identification of individual compounds; gradient elution	mg/g of individual compounds	Expensive; requires standards and expertise	[16,17,45]
Apocarotenoids (crocin, picrocrocin, safranal)	HPLC/GC-MS/UPLC-MS/MS	Separation and detection of volatile/non-volatile compounds	Compound-specific detection; optimized extraction	Compound-specific (µg/g, ng/mL)	Rarely applied to petals; mainly used for stigmas	[34,49,53,54]

**Table 5:** Comparisons of analytical methods for carotenoids, crocin, picrocrocin, and safranal in saffron stigmas and petals.

Compound	Matrix	Method	Key Conditions/ Values	What it Measures	Limitations	Reference
Total carotenoids (TCC)	Stigmas	UV–Vis spectrophotometry	λ ≈ 450 nm; up to 546.55 μg/g	Total carotenoids	Non-specific; no separation of crocin/crocetin	[29,30]
	Petals/floral parts	Solvent extraction + UV–Vis	Acetone + petroleum ether; λ ≈ 450 nm	Total carotenoids	Pigment interference (anthocyanins); solvent-dependent recovery	[34]
Crocin (individual)	Stigmas	HPLC–UV/HPLC–DAD	Separation of multiple crocin isomers	Specific crocin profile	Requires standards; time-consuming	[55,57]
	Stigmas	UPLC-MS/MS	~111–128 ng/mL	Highly sensitive crocin quantification	Expensive; advanced instrumentation	[53]
	Stigmas	LC-MS	Internal standard (2-nitroaniline)	Accurate crocin quantification	Complex sample prep	[58]
	Petals	Rarely quantified	—	—	Major data gap; not routinely analyzed	[59]
Crocetin	Stigmas	HPLC	Crocetin-based quantification	Total crocin (via conversion)	Requires hydrolysis step	[60]
	Petals	Not reported	—	—	Major data gap; not routinely analyzed	—
Picrocrocin	Stigmas	HPLC/HPTLC	Detection at ~254 nm	Bitter compound quantification	Degradation during processing	[57]
	Stigmas	GC-MS	Volatile fraction 0.4–1.3%	Aroma precursors	Instability; processing-dependent	[34,54]
	Petals	Not reported	—	—	Major data gap; not routinely analyzed	—
Safranal	Stigmas	GC-MS/HS-GC-MS	Volatile analysis; 0.4–1.3%	Aroma compound	Loss during extraction; volatility issues	[34,54]
	Stigmas	GC-MS (advanced extraction)	Ultrasound / optimized extraction	Improved volatile recovery	Method-sensitive	[61]
	Petals	Rarely analyzed	—	—	Major data gap; not routinely analyzed	—

**Table 6:** Mineral composition of saffron petals reported in studies.

Mineral	Type	Concentration Range	Matrix/Origin	Key Observations	Reference
K	Macro-mineral	542 mg/100 g	Iranian petals	Most abundant mineral; consistently dominant across studies	[33]
K	Macro-mineral	~1500 mg/100 g (dry weight)	Spanish floral by-products	Significantly higher values; influenced by origin and processing	[18]
K	Macro-mineral	~97.5 ppm	Other petal samples	Lower reported values depending on analytical method and matrix	[5]
Ca	Macro-mineral	486.25 mg/100 g	Iranian petals	Second-most abundant mineral	[33]
Ca	Macro-mineral	112.60–415.20 mg/100 g	Spanish petals	High variability attributed to soil and geo-climatic conditions	[18]
P	Macro-mineral	209.90 mg/100 g	Iranian petals	Moderate levels; contributes to nutritional value	[33]
Na	Macro-mineral	25.75 mg/100 g	Iranian petals	Low concentration; nutritionally favorable (low sodium content)	[33]
Fe	Micro-mineral	Reported (not quantified here)	Various petal studies	Essential micronutrient; contributes to functional properties	[33]
Zn	Micro-mineral	Reported (not quantified here)	Various petal studies	Present in trace amounts	[33]
Mg	Micro-mineral	Reported (not quantified here)	Various petal studies	Important for metabolic functions	[33]

**Table 7:** Analytical techniques and applications for mineral determination in saffron petals.

Minerals/ Elements	Analytical Technique	Application	Reference
Multi-element profile (K, Ca, Fe, Mg, trace metals)	ICP-MS + Stable Isotope Ratio Analysis/ICP-OES	Geographical discrimination and authenticity assessment of saffron	[65,66,67]
Macro- and microelements	ICP-MS/ICP-OES/AAS	Nutritional characterization and evaluation of saffron petals as ingredient for animal feed	[33,65,66,67]
Trace and heavy metals (Pb, Cd, Zn, Cu, Ni, Mn, Fe)	ICP-MS/ICP-OES/AAS	Elemental fingerprinting and geographical-origin discrimination	[62,64,65]
Macro- and microelements in food matrices	ICP-MS/ICP-OES/AAS	Nutritional profile of bakery products enriched with saffron petals	[18,65]

**Table 8:** Saffron petal drying methods: comparison of drying methods and their impacts on the compositional profiles of saffron petals.

Method	Reported Parameters	Impact on Compositional Profile	Advantages	Disadvantages	Reference
Oven-Drying	40 °C (24 h) to 60 °C (4–8 h)	Higher crocin content, (at 60 °C)	Excellent for extracting minor compounds like crocins and safranal at 60 °C.	Long exposures at 60 °C can degrade sensitive anthocyanins.	[39,68]
Vacuum Evaporation	50 °C (approx. 2 h/2 cycles)	High antioxidant activity (flavonoids)	Fastest method; superior for isolating flavonoids, protecting anthocyanins from oxygen degradation.	Requires specialized equipment (vacuum pump/condenser).	[18]
Freeze-Drying (Lyophilization)	−50 °C for 24–48 h	High antioxidant activity (flavonoids)	Maximum retention of anthocyanins, flavonoids; best for maintaining original morphology.	Most expensive method; lower yield for crocins compared to heat-based methods.	[18]
Traditional Air-Drying	Room temp, airy space (2–4 days)	High content of anthocyanin	Low cost; traditional for tea or simple preservation.	Risk of mold growth in humid climates and enzymatic biodegradation.	[69]
Microwave Drying	Low power (e.g., 400–600 W) for 3–6 min	High content of anthocyanin	Highly efficient and rapid; can preserve aroma well at low power.	High power (900 W+) causes significant thermal degradation of active components.	[68,69]

**Table 9:** Solvent impact on saffron petals extracts used as natural preservatives.

Parameter	Aqueous (Water)	Ethanolic (70–80%)	Reference
Yield	High (60–70%)	Moderate (45–58%)	[7,24]
Phenolic Content	Lower	Highest	[16,17]
Antioxidant Power	Weak to Moderate	Strong	[45]
Bacterial Sensitivity	More effective on Gram-positive bacteria	More effective on Gram-positive bacteria	[16]

**Table 10:** Comparison between extraction methods used for saffron petals.

Method	Typical Speed	Anthocyanin Yield	Phenolic/Flavonoid Yield	Reported Antioxidant Effect	References
Maceration	Slow, conventional	Anthocyanins up to 413.30 mg G3G/100 g DW	Total phenolics ~1127.94 mg GAE/100 g DW in dried petals	High antioxidant responses (FRAP/ABTS/DPPH) reported for extracts from dried petals	[1,31]
Ultrasound-assisted extraction (UAE)	Faster (often ~half time of maceration)	High anthocyanin recovery; e.g., 93.43 ± 4.67 mg/g dry plant with 50:50 EtOH/H_2_O in one study	Phenolic and flavonoid yields comparable or superior to maceration, depending on solvent	Generally similar or improved antioxidant activity vs. maceration when using aqueous/low-MeOH solvents	[1,21]
Microwave-assisted extraction (MAE)	Rapid, promising green option	Quantitative comparisons limited in available texts; MAE proposed as effective for bioactives	Quantitative MAE yields not fully reported in supplied abstracts	Insufficient evidence in supplied papers for direct antioxidant comparisons.	[26]
Subcritical water extraction (SWE)	Fast, temperature-dependent	SWE variants gave strong results; best SWE conditions included high EtOH at 125 °C in one study	SWE under optimized conditions provided competitive phenolic yields versus other GETs	Antioxidant outcomes depend on conditions; direct cross-method antioxidant comparisons are limited	[21]
Deep eutectic solvent extraction (DESE)	Variable by DES composition	Anthocyanins were lower with DESE overall; specific DES (choline chloride: butane-1,4-diol) gave anthocyanins ~16.0 ± 0.80 mg/g dp	DESE produced the highest flavonoid totals in the tested solvent sets (110.95–73.25 mg/g dp ranges)	Antioxidant implications follow the phenolic profile but direct antioxidant assay comparisons are not fully reported	[21]

**Table 11:** UV-C/UV-B impact: a comparison between saffron petals and stigmas.

Impact	Saffron Stigmas (Spice)	Saffron Petals (By-Product)	Reference
Primary Pigment Affected	Crocins (Red/Yellow carotenoids).	Anthocyanins (Purple flavonoids).	[71]
UV-C Impact on Color	Degrades crocin content by 29–30% after 180 min of exposure.	Traditional sun-drying maintains high stability, but intense UV-C can cause rapid browning.	[71]
Antioxidant Response	UV exposure can increase phenolic and flavonoid content as a defensive “UV-absorbent” response.	Petals naturally have higher antioxidant capacity (IC50) than stigmas, even without stress.	[71]
Aroma/Flavor Impact	Significant loss of safranal (aroma), by up to 45%.	Contains lower concentrations of safranal; degradation of kaempferol derivatives.	[71]
Harvest Protection	Buds must be harvested before dawn; even a few hours of sun reduce coloring strength.	Petals are highly susceptible to rapid deterioration and oxidation if left in the field post-bloom.	[71]

**Table 12:** Compositional profile of bakery products enriched with saffron petals.

Component	Parameter	Saffron Petals (Bakery Products)	Addition Type	Bakery Product	Reference
Proximate composition	Protein (%)	~8–12 (slight variation)	Dried petals (2.5–10%)	Bread	[9]
	Carbohydrates (%)	~60–70 (dominant fraction)	Dried petals	Bread	[9]
	Lipids (%)	Recipe-dependent (minor variation)	Dried petals	Bread	[9]
	Fiber (%)	Increase up to 25–30%	Dried petals	Bread	[9]
	Moisture (%)	Slight variation	Dried petals	Bread	[9]
	Ash (%)	Slight increase	Dried petals	Bread	[9]
Bioactive compounds	Total phenolic content	Increase up to ~2×	Dried petals	Bread	[9]
	Flavonoids/Anthocyanins	Petal-derived compounds retained after processing	Petal extract/fermented system	Bread	[12]
	Antioxidant activity	Increased (relative improvement)	Dried petals	Bread	[9]
	Antifungal activity	Up to 44.33% inhibition	Petal extract (fermented system)	Bread	[12]
	Bioaccessibility	Phenolics stable after digestion	Dried petals	Bread	[9]
Minerals	K (mg/100 g)	162 → 277–289	Dried petals	Bread	[9]
	Ca (mg/100 g)	~90–95	Dried petals	Bread	[9]
	Mg (mg/100 g)	~40–50	Dried petals	Bread	[9]
	Fe (mg/100 g)	~2 → 15–18	Dried petals	Bread	[9]
	Na (mg/100 g)	Minor variation	Dried petals	Bread	[9]
	Trace elements (Zn, Cu, Mn)	Not reported	—	—	—
	Heavy metals (Pb, Cd, Hg)	Not reported in bakery products	—	—	—

**Table 13:** Analytical methods used for proximate analysis in bakery products enriched with saffron petals.

Parameter	Bakery Product	Method	Conditions/Details	Reference
Moisture	Bread (wheat, spelt)	Oven drying (AOAC)	105 ± 5 °C until constant weight	[9]
	Bread (sourdough)	Oven drying (AOAC)	Standard method	[12]
Ash	Bread	Dry ashing (AOAC)	Incineration at 550 ± 25 °C	[9]
	Bread	Dry ashing (AOAC)	Standard incineration	[12]
Protein	Bread	Kjeldahl method (AOAC)	N × 6.25 conversion factor	[9]
	Bread	Kjeldahl method (AOAC)	Standard method	[12]
Lipids	Bread	Soxhlet extraction (AOAC)	Organic solvent extraction	[9]
	Bread	Soxhlet extraction (AOAC)	Standard method	[12]
Carbohydrates	Bread	By difference	100 − (moisture + protein + fat + ash)	[9]
	Bread	By difference	Standard calculation	[12]
Fiber	Bread	Enzymatic–gravimetric (AOAC)	Total dietary fiber determination	[9]

**Table 14:** Summary of studies on saffron petal incorporation in bakery products.

Inclusion Method	Bakery Product	Effect on Final Product	Reference
Powder (2.5–10%)	Wheat and spelt bread	↑ Dietary fiber (up to +25–30%); ↑ minerals (K: 277–289 mg/100 g; Fe: 15–18 mg/100 g); ↑ antioxidant activity; ↓ pH (~5.2); ↑ acidity (up to 0.28%); altered texture (↑ hardness, ↓ gluten strength); color changes; moderate sensory impact at higher levels	[9]
Extract (aqueous, fermented system)	Wheat bread with mung bean sourdough	↑ Antifungal activity (up to 44.33% inhibition of A. niger); ↑ shelf life (~4 days mold-free); improved texture (hardness ↓ to 10.21 N; porosity ↑ to 16.16%)	[12]
Extract (direct addition)	Wheat bread	↑ Bioactive compounds; slight negative impact on structure (↑ hardness, ↓ porosity/volume); color modification (violet/gray tones); minimal effect on proximate composition	[12]

↑ increase; ↓ decrease.

**Table 15:** Analytical methods for bioactive compounds in saffron petal-enriched bakery products, and their applicability.

Method	Measures	Use in Bakery Products	Advantages	Limitations in Bakery Matrices	Reference
Folin–Ciocalteu	Total phenolics (GAE)	Commonly used	Simple, rapid, widely applied	Non-specific; affected by sugars, proteins, Maillard products → overestimation	[9,49]
AlCl_3_ assay	Total flavonoids (QE)	Occasionally used	Simple, low-cost	Limited specificity; matrix interference	[34]
DPPH	Radical scavenging activity	Commonly used	Fast, widely applied	Strong matrix and solvent effects; not compound-specific	[9]
ABTS	Antioxidant capacity	Used	Works for hydrophilic & lipophilic compounds	Matrix-dependent variability	[9]
FRAP	Reducing power	Used	Simple, reproducible	Measures reducing power, not true radical scavenging; affected by non-phenolics	[9]
UV–Vis (pigments)	Total pigments	Rarely applied in bakery products	Rapid, low-cost	Poor specificity; interference from co-extracted compounds	[29,30,34]
HPLC (DAD/UV)	Individual phenolics/flavonoids	Rarely applied in bakery products (mainly petal studies)	High specificity and accuracy	Complex sample preparation; underutilized in bakery matrices	[16,17,44]
LC-MS/ UPLC-MS/MS	Detailed compound profile	Not applied in bakery products	Very high sensitivity and selectivity	Expensive; limited to raw material characterization	[15,44]

**Table 16:** Mineral composition of bakery products enriched with saffron petals.

Type	Mineral	Product/Matrix	Concentration	Addition	Reference
Macrominerals	Ca	Wheat bread	90–95 mg/100 g	Dried petals (2.5–10%)	[9]
	K	Wheat bread	162 → 277–289 mg/100 g	Dried petals	[9]
	Mg	Wheat bread	40–50 mg/100 g	Dried petals	[9]
	Na	Wheat bread	Minor variation (not significant)	Dried petals	[9]
Microminerals	Fe	Wheat bread	~2 → 15–18 mg/100 g	Dried petals	[9]
	Zn, Cu, Mn	Bakery products	Not reported	—	—
Toxic elements	Pb	Raw petals (reference for safety)	0.18–0.31 mg/kg	Petals	[73]
	Cd	Raw petals	0.04–0.09 mg/kg	Petals	[73]
	Hg	Raw petals	0.18–0.25 mg/kg	Petals	[73]
	As	Raw petals	0.21–0.40 mg/kg	Petals	[73]

**Table 17:** Comparative analytical methods for textural parameters of bakery products enriched with saffron petals.

Inclusion Method	Bakery Product	Parameter Measured	Effect Compared to Control	Key Values	Method	Reference
Dried saffron petal powder (2.5–10%)	Wheat/spelt bread	Hardness	Increased with concentration	Higher firmness at ≥5–10%	TPA	[9]
		Elasticity/cohesiveness	Decreased	Reduced gluten network strength	TPA	[9]
		Volume/gas retention	Reduced	Lower loaf volume at higher levels	Physical measurement	[9]
		Crumb structure	Altered (denser)	Reduced porosity	Image/structural analysis	[9]
Saffron petal extract (direct addition)	Wheat bread	Hardness	Increased	~14.86 N	TPA	[12]
		Porosity	Decreased	~13.5%	Image analysis	[12]
Saffron petal extract + fermentation	Wheat bread (sourdough)	Hardness	Improved vs. extract-only	10.21 N	TPA	[12]
		Porosity	Increased	16.16%	Image analysis	[12]
		Shelf-life	Improved	~4 days mold-free	Microbial observation	[12]

**Table 18:** Sensory properties of bakery products enriched with saffron petals.

Inclusion Method	Bakery Product	Sensory Parameter	Effect Compared to Control	Key Observations	Method	Reference
Dried saffron petal powder (2.5–10%)	Wheat/spelt bread	Overall acceptability	Optimal at low–moderate levels; decreased at high levels	Best scores at 2.5–5%; decline at 10% due to texture and color	Sensory panel (hedonic scale)	[9]
		Taste	Slight bitterness at higher concentrations	Acceptable at ≤5%; bitterness at ≥10%	Sensory panel	
		Aroma	Mild floral notes	Generally acceptable, not dominant	Sensory panel	
		Texture perception	Decreased softness	Firmer crumb perceived negatively at high levels	Sensory panel	
		Color	Altered (yellow–reddish/violet tones)	Acceptable at low levels; too intense at high levels	Visual sensory evaluation	
Saffron petal extract (direct addition)	Wheat bread	Overall acceptability	Slight decrease	Lower scores due to hardness and reduced porosity	Sensory panel	[12]
		Texture perception	Increased acceptability	Harder texture negatively perceived	Sensory panel	
Saffron petal extract + fermentation	Wheat bread (sourdough)	Overall acceptability	Improved vs. extract-only	Acceptable sensory profile restored	Sensory panel	[12]
		Aroma/flavor	Improved	Enhanced flavor complexity due to fermentation	Sensory panel	
		Texture perception	Improved	Softer crumb, improved mouthfeel	Sensory panel	

**Table 19:** Pharmacological effects of saffron petals (P) or extracts (E).

Pharmacological Effect	Type of Study	Petal/Extract	Results	Reference
Antioxidant	In vitro B16 cells ^1^	Extract	Prevent diseases caused by oxidative stress due to high content of kaempferol-3-O-sophoroside and quercetin-3-O-sophoroside (quercetin, 4.03 ± 0.33 mg g^−1^ DW; kaempferol, 47.80 ± 0.60 mg g^−1^ DW).	[81]
Antibacterial/Antimicrobial activity	In vitro	Extract	Effects depending on the type of extract and doses. They are effective against Escherichia coli, Salmonella typhimurium, Staphylococcus aureus, and Listeria monocytogenes.	[16,17,26]
Anti-inflammatory	In Vitro RAW 264.7 cells	Extract	Assessing the generation of reactive oxygen species. Determination of NO Production.	[1,82]
	Animal experiment mice- induced inflammation model	Extract	Anti-inflammatory effect by regulating autophagy and the NLRP3–NF-κB pathway.	[78,83]
Gynecological disease; polycystic ovary syndrome	Animal experiment (mice)	Extract petal anthocyanin	Improved dysregulation of ovarian steroids, steroidogenic, antioxidant enzymes and inflammatory markers.	[78]
Anti-diabetic	In vitro	Extracts using CE ^2^, MAE ^2^ and UAE ^2^	α-amylase inhibition assay; antidiabetic activity: 81% (CE), 75% (MAE), 71% (UAE).	[5]
Antidepressant	Animal experiment	Extract	Kaempferol; flavonoid of the tepals was reported to have antidepressant activity on mice and rats.	[56]
Anticancer	In vitro	Extract	Assessing different extracts; cytotoxic activity against liver cancer cells.	[63]
Anti-dyslipidemia	Double-blinded randomized clinical trial	Petal pills Hydroalcoholic extract	Reduced blood serum lipid profile, urea and CR. Prevention of dyslipidemia, cardiovascular disorders.	[84]
Anti-spasmodic	Animal experiment	Extract (hydroethanolic)	Significant antispasmodic effect against contraction generated by CCh (carbamylcholine) (10–6 M) and KCl (25 mM), depending on the dose administrated.	[47]
Neuroprotective activity	Extracts Animal	Extract	Neuropsychiatric and age-related diseases. Bioactive compounds, kaempferol, are effective in reducing neuroinflammation and protecting neurons from damage. Potential as a low-cost alternative for managing conditions (Alzheimer, Parkinson).	[47,85]
Antihypertensive	Animal experiment; In vitro	Extract (kaempferol)	Reduce ROS, increase antioxidant indicators level (in vitro). Ameliorates induced cardiovascular damage (mice).	[85]
Anti-tyrosinase activity	Powder	Saffron petals powder	Exhibit anti-tyrosinase activity by inhibiting tyrosinase, the copper-dependent, rate-limiting enzyme in melanogenesis.	[16]

^1^ B16 melanoma is a murine tumor cell line used for research as a model for human skin cancers. ^2^ Conventional extraction (CE), Microwave-assisted extraction (MAE) and Ultrasound-assisted extraction (UAE).

## Data Availability

No new data were created in this study. The data supporting the findings were obtained from publicly available published literature and are reported within the article.

## Abbreviations

The following abbreviations are used in this manuscript:

AAS	Atomic Absorption Spectroscopy
ABTS	2,2′-Azino-bis (3-ethylbenzothiazoline-6-sulfonic acid)
AHP	Analytic Hierarchy Process
AOAC	Association of Official Analytical Chemists
CCh	Carbamylcholine
CE	Conventional Extraction
CIE	Commission Internationale de l’Éclairage
DESE	Deep Eutectic Solvent Extraction
DNA	Deoxyribonucleic Acid
DPPH	2,2-Diphenyl-1-picrylhydrazyl
DW	Dry Weight
EtOH	Ethanol
F–C	Folin–Ciocalteu
Fp	Nitrogen-to-Protein Conversion Factor
FRAP	Ferric Reducing Antioxidant Power
GAE	Gallic Acid Equivalents
GC-MS	Gas Chromatography–Mass Spectrometry
HPLC	High-Performance Liquid Chromatography
HPLC-ESI-MS	High-Performance Liquid Chromatography–Electrospray Ionization Mass Spectrometry
HPLC-PDA	High-Performance Liquid Chromatography with Photodiode Array
HS-GC-MS	Headspace Gas Chromatography–Mass Spectrometry
IC_50_	Half Maximal Inhibitory Concentration
ICP-MS	Inductively Coupled Plasma Mass Spectrometry
ICP-OES	Inductively Coupled Plasma Optical Emission Spectrometry
ISO	International Organization for Standardization
KCl	Potassium Chloride
LC-MS	Liquid Chromatography–Mass Spectrometry
LD_50_	Lethal Dose for 50% of subjects
MAE	Microwave-Assisted Extraction
MCDA	Multi-Criteria Decision Analysis
MCDM	Multiple-Criteria Decision-Making
MeOH	Methanol
MIC	Minimum Inhibitory Concentration
NF-κB	Nuclear Factor Kappa-Light-Chain-Enhancer of Activated B Cells
NIR	Near-Infrared Spectroscopy
NLRP3	NOD-like Receptor Family Pyrin Domain Containing 3
NO	Nitric Oxide
NMR	Nuclear Magnetic Resonance
NPN	Non-Protein Nitrogen
ORAC	Oxygen Radical Absorbance Capacity
PBS	Phosphate-Buffered Saline
POD	Protected Designation of Origin
PRISMA	Preferred Reporting Items for Systematic Reviews and Meta-Analyses
QDA	Quantitative Descriptive Analysis
QE	Quercetin Equivalents
ROS	Reactive Oxygen Species
SWE	Subcritical Water Extraction
TCATA	Temporal Check-All-That-Apply
TCC	Total Carotenoid Content
TFC	Total Flavonoid Content
TPA	Texture Profile Analysis
TOPSIS	Technique for Order of Preference by Similarity to Ideal Solution
TPC	Total Phenolic Content
UAE	Ultrasound-Assisted Extraction
UPLC-MS/MS	Ultra Performance Liquid Chromatography–Tandem Mass Spectrometry

## Appendix A

**Table A1:** Proposed roadmap of future studies for the development of bakery products containing saffron petals.

Research Objective	Recommended Methodology	Primary Target Compounds	Outcome
Antioxidant Power	FRAP/ABTS/DPPH Assays	Polyphenols, Flavonoids	Trolox Equivalent (TE): Comparative antioxidant capacity relative to a standard.
Mineral Bioavailability	ICP-MS ^1^; ICP-OES ^2^	Al (Aluminum), As (Arsen), B (Bohr), Ca (Calcium), Cd (Cadmium), Cr (Cronium), Co (Cobalt), Cu (Copper), Fe (Iron), Ga (Gallium), In (Indium), P (Phosphorus), K (Potassium), Mg (Magnesium), Mn (Mangan), Mo (Molibden), Na (Natrium), Ni (Nichel), Pb (Lead), Sr (Strontium), Zn (Zinc), Se (Selenium), Hg (Mercury)	Solubility Ratio: The percentage of minerals released from the fiber matrix.
Fingerprinting	LC-MS/MS ^3^ (Liquid Chromatography–Mass Spectrometry)	Quercetin, Isorhamnetin, Specific Flavonols	Metabolic Profile: Precise chemical “ID card” for petal-enriched vs. control bread.
Thermal Stability	Arrhenius Kinetic Modeling (during baking)	Anthocyanins (Malvidin/Delphinidin Derivatives)	Half-life (t1/2): Time/temp at which 50% of pigments degrade.
Nutritional Digestion	INFOGEST 2.0 (In vitro static digestion)	Kaempferol glycosides, Anthocyanins, Total Phenolics	Bioaccessibility Index (BI%): Amount of compound available for absorption after digestion.
Sensory Evaluation	Temporal Check-All-That-Apply (TCATA)	Volatile Organic Compounds, Terpenes	Flavor Persistence: Tracking “floral” vs. “bitter” notes during the chewing process.
Clinical Efficacy	Clinical trials on bakery products containing saffron petals or extracts (including encapsulated)	Plasma metabolites (phase II) and free aglycones (blood plasma) and peak concentration; Urine: Total Polyphenol Excretion (TPE); TPE normalized by Creatinine; HPLC-MS-Specific metabolite profiling; Plasma kaempferol specific metabolites after eating bakery with saffron petals/extracts	Accurate daily absorption and immediate bioavailability. Large-scale clinical monitoring. Tracking specific food sources. Kaempferol bioavailability depending on food matrix.

^1^ ICP-MS Inductively Coupled Plasma Mass Spectrometry. ^2^ ICP-OES Inductively Coupled Plasma Optical Emission Spectrometry. ^3^ LC-MS/MS Liquid Chromatography–Mass Spectrometry.

## References

[1] Kumar R., Subba R., Chettri A., Rai S.K., Bag N., Chhetri D.R., Khare A., Sharma L., Sharma S.S. (2025). Saffron (*Crocus sativus* L.) cultivation under organic regime in Sikkim Himalaya and prevalence of conditions conducive for corm multiplication. Sci. Rep..

[2] Lachguer K., El Fels L., El Hajjaji S., El Hamdani M., Khouili M., Ounine K. (2022). Major phytochemical compounds, in vitro antioxidant, antibacterial, and antifungal activities of six aqueous and organic extracts of *Crocus sativus* L. flower waste. Waste Biomass Valorization.

[3] Ordoudi S.A., Tsimidou M.Z. (2024). Building a database for the quality characteristics of the Protected Designation of Origin saffron Krokos Kozanis, considering international trade requirements. Explor. Foods Foodomics.

[4] Senizza B., Rocchetti G., Ghisoni S., Busconi M., De Los Mozos Pascual M., Fernandez J.A., Lucini L., Trevisan M. (2019). Identification of phenolic markers for saffron authenticity and origin: An untargeted metabolomics approach. Food Res. Int..

[5] Gull A., Masoodi F.A., Rizwan D., Bakshi R.A., Gani A., Wani I.A. (2025). Valorising saffron petal, a study on nutritional potential; phytochemical, antimicrobial and antidiuretic activity of a promising agro by product. Waste Biomass Valorization.

[6] Serrano-Díaz J., Sánchez A.M., Maggi L., Martínez-Tomé M., García-Diz L., Murcia M.A., Alonso G.L. (2012). Increasing the applications of *Crocus sativus* flowers as natural antioxidants. J. Food Sci..

[7] Shanker M.A., Puthiyedath A., Rana S.S. (2025). Floral bio-residues of saffron: A potential source of valuable components; extraction and application. Discov. Sustain..

[8] Cerdá-Bernad D., Clemente-Villalba J., Valero-Cases E., Pastor J.-J., Frutos M.-J. (2022). Novel insight into the volatile profile and antioxidant properties of *Crocus sativus* L. flowers. Antioxidants.

[9] Cerdá-Bernad D., Frutos M.J. (2023). Saffron floral by-products as novel sustainable vegan ingredients for the functional and nutritional improvement of traditional wheat and spelt breads. Foods.

[10] Mohaqiq Z., Moossavi M., Hemmati M., Kazemi T., Mehrpour O. (2020). Antioxidant properties of saffron stigma and petals: A potential therapeutic approach for insulin resistance through an insulin-sensitizing adipocytokine in high-calorie diet rats. Int. J. Prev. Med..

[11] Turcov D., Barna A.S., Ciuperca O.T.A., Trifan A., Puitel A.C., Suteu D. (2022). Valorization of bioactive compounds from residual saffron biomass (*Crocus sativus* L.) to obtain high value added dermato-cosmetic products. BioResources.

[12] Ziaee Rizi A., Sadeghi A., Feizi H., Jafari S.M., Purabdolah H. (2024). Evaluation of textural, sensorial and shelf-life characteristics of bread with saffron petal extract. Iran. J. Food Sci. Ind..

[13] Page M.J., McKenzie J.E., Bossuyt P.M., Boutron I., Hoffmann T.C., Mulrow C.D., Shamseer L., Tetzlaff J.M., Akl E.A., Brennan S.E. (2021). The PRISMA 2020 statement: An updated guideline for reporting systematic reviews. BMJ.

[14] (2010). Saffron (*Crocus sativus* L.)—Part 2: Test Methods.

[15] Zhou L., Cai Y., Yang L., Zou Z., Zhu J., Zhang Y. (2022). Comparative Metabolomics Analysis of Stigmas and Petals in Chinese Saffron (*Crocus sativus*) by Widely Targeted Metabolomics. Plants.

[16] Naim N., Fauconnier M.-L., Ennahli N., Tahiri A., Baala M., Madani I., Ennahli S., Lahlali R. (2022). Chemical Composition Profiling and Antifungal Activity of Saffron Petal Extract. Molecules.

[17] Naim N., Bouymajane A., El Majdoub Y.O., Ezrari S., Lahlali R., Tahiri A., Ennahli S., Vinci R.L., Cacciola F., Mondello L. (2023). Flavonoid Composition and Antibacterial Properties of *Crocus sativus* L.. Petal Extracts. Mol..

[18] Cerdá-Bernad D., Valero-Cases E., Pérez-Llamas F., Pastor J.J., Frutos M.J. (2023). Underutilized *Crocus Sativus* L. Flowers: A Hidden Source of Sustainable High Value-Added Ingredients. Plant Foods Hum. Nutr..

[19] Belyagoubi-Benhammou N., Belyagoubi L., Loukidi B., Mir M.A., Assadpour E., Boudghene-Stambouli M., Kharazmi M.S., Jafari S.M. (2024). Bioactivity and applications of saffron floral bio-residues (tepals): A natural by-product for the food, pharmaceutical, and cosmetic industries. Crit. Rev. Food Sci. Nutr..

[20] Melnyk J.P., Wang S., Marcone M.F. (2010). Chemical and biological properties of the world’s most expensive spice: Saffron. Food Res. Int..

[21] Stelluti S., Caser M., Demasi S., Scariot V. (2021). Sustainable Processing of Floral Bio-Residues of Saffron (*Crocus sativus* L.) for Valuable Biorefinery Products. Plants.

[22] Righi V., Parenti F., Tugnoli V., Schenetti L., Mucci A. (2015). *Crocus sativus* Petals: Waste or Valuable Resource? The Answer of High-Resolution and High-Resolution Magic Angle Spinning Nuclear Magnetic Resonance. J. Agric. Food Chem..

[23] Dina E., Cheilari A., Karamani D., Mitsopoulos V., Diamanti I., Fokialakis N., Aligiannis N. (2025). Investigating the Preservation and Utilization of the Saffron (*Crocus sativus* L.) Sorting By-Product (Tepals). Plants.

[24] Masala V., Jokić S., Aladić K., Molnar M., Tuberoso C.I.G. (2024). Exploring Phenolic Compounds Extraction from Saffron (*C. sativus*) Floral By-Products Using Ultrasound-Assisted Extraction, Deep Eutectic Solvent Extraction, and Subcritical Water Extraction. Molecules.

[25] Slimani C., Fadil M., Rais C., El-Hanafi L., Benjelloun M., Shin H.-M., Giesy J.P., Lazraq A., Abulmeaty M.M., Almutairi K.M. (2025). Extraction of natural antioxidants from Moroccan saffron (*Crocus sativus* L.) using ultrasound-assisted extraction: An optimization approach with box-behnken design. Ultrason. Sonochemistry.

[26] Hosseini S.R., Ghavam M. (2025). Saffron petal waste as a source of natural antimicrobials: Comparative analysis of extraction methods. Discov. Appl. Sci..

[27] Jadouali S., Atifi H., Bouzoubaa Z., Majourhat K., Gharby S., Achemchem F., Elmoslih A., Laknifli A., Mamouni R. (2018). Chemical characterization, antioxidant and antibacterial activity of Moroccan *Crocus sativus* L. petals and leaves. J. Mater. Environ. Sci..

[28] Amanpour A., Soltani M., Lipan L., Garcia-Garví J.M., Hernández-García F., Carbonell-Barrachina Á.A., Nadal E.S. (2024). Comparative study on nutraceutical and sensorial characteristics of saffron (*Crocus sativus* L.) cultivated in Iran, Spain, and Türkiye. J. Sci. Food Agric..

[29] Baba S.A., Malik A.H., Wani Z.A., Mohiuddin T., Shah Z., Abbas N., Ashraf N. (2015). Phytochemical analysis and antioxidant activity of different tissue types of *Crocus sativus* and oxidative stress alleviating potential of saffron extract in plants, bacteria, and yeast. S. Afr. J. Bot..

[30] Bakshi R.A., Sodhi N.S., Wani I.A., Khan Z.S., Dhillon B., Gani A. (2022). Bioactive constituents of saffron plant: Extraction, encapsulation and their food and pharmaceutical applications. Appl. Food Res..

[31] Porto C.D., Natolino A. (2018). Extraction kinetic modelling of total polyphenols and total anthocyanins from saffron floral bio-residues: Comparison of extraction methods. Food Chem..

[32] Jadouali S.M., Atifi H., Mamouni R., Majourhat K., Bouzoubaâ Z., Laknifli A., Faouzi A. (2019). Chemical characterization and antioxidant compounds of flower parts of Moroccan *Crocus sativus* L.. J. Saudi Soc. Agric. Sci..

[33] Fahim N.K., Janati S.S.F., Feizy J. (2012). Chemical composition of agriproduct saffron (*Crocus sativus* L.) petals and its considerations as animal feed. GIDA—J. Food.

[34] Fardaghi A., Es-haghi A., Feizy J., Lakshmipathy R. (2021). Antioxidant capacity and chemical composition of saffron flowers. J. Food Bioprocess Eng..

[35] AOAC International (1995). Official Methods of Analysis.

[36] Emadi B., Saiedirad M.H. (2011). Moisture-dependent physical properties of saffron flower. J. Agric. Sci. Technol..

[37] Jadouali S.M., Atifi H., Mamouni R., Majourhat K., Bouzoubaa Z., Gharby S. (2022). Composition of saffron by-products (*Crocus sativus*) in relation to utilization as animal feed. Agric. Sci. Dig..

[38] Pontin M., Poggi L., Berli F., Bolcato L., Piccoli P., Fontana A. (2026). Characterisation of saffron floral bio-residues phenolic compounds under different drying treatments: From agricultural waste to bio-based ingredients. Food Chem. Adv..

[39] Criado-Navarro I., Barba-Palomeque F., Pérez-Juan P., Ledesma-Escobar C.A., Priego-Capote F. (2024). Drying of saffron petals as a critical step for stabilization prior to extraction of bioactive compounds. Foods.

[40] Locatelli I., Pedrali D., Grassi S., Buratti S., Giorgi A., Giupponi L. (2025). Progress in quality assessment of Italian saffron. Sci. Rep..

[41] Escobar-Talavera J.F., Martínez-Navarro M.E., Alonso G.L., Sánchez-Gómez R. (2024). Determination of saffron flower metabolites by near-infrared spectroscopy for quality control. Horticulturae.

[42] Alighaleh P., Pakdel R., Ghanei Ghooshkhaneh N., Einafshar S., Rohani A., Saeidirad M.H. (2023). Detection and classification of saffron adulterants by Vis-NIR imaging, chemical analysis, and soft computing. Foods.

[43] Mariotti F., Tomé D., Mirand P.P. (2008). Converting nitrogen into protein—Beyond 6.25 and Jones’ factors. Crit. Rev. Food Sci. Nutr..

[44] Li X., Song J., Tan J., Zhang D., Guan Y., Geng F., Yang M., Pei J., Ma H. (2024). “Plant golden” *Crocus sativus*: Qualitative and quantitative analysis of major components in stigmas and petals and their biological activity in vitro. J. Pharm. Biomed. Anal..

[45] Maestre-Hernández A.-B., Vicente-López J.-J., Pérez-Llamas F., Candela-Castillo M.-E., García-Conesa M.-T., Frutos M.-J., Cano A., Hernández-Ruiz J., Arnao M.B. (2023). Antioxidant activity and phenolic content in saffron bio-residues. Processes.

[46] Moratalla-López N., Bagur M.J., Lorenzo C., Martínez-Navarro M.E., Salinas M.R., Alonso G.L. (2019). Bioactivity and bioavailability of the major metabolites of *Crocus sativus* L. flower. Molecules.

[47] Qadir M.M., Nissar J., Dar A.H., Ganaie T.A., Bashir M. (2024). Insights into phytochemistry and bioactive potential of saffron (*Crocus sativus* L.) petals. Future Postharvest Food.

[48] Ruggieri F., Maggi M.A., Rossi M., Consonni R. (2023). Comprehensive Extraction and Chemical Characterization of Bioactive Compounds in Tepals of *Crocus sativus* L.. Molecules.

[49] Everette J.D., Bryant Q.M., Green A.M., Abbey Y.A., Wangila G.W., Walker R.B. (2010). Thorough Study of Reactivity of Various Compound Classes toward the Folin−Ciocalteu Reagent. J. Agric. Food Chem..

[50] Lamuela-Raventós R.M., Apak R., Capanoglu E., Shahidi F. (2018). Folin–Ciocalteu method for the measurement of total phenolic content and antioxidant capacity. Measurement of Antioxidant Activity & Capacity.

[51] Dominguez-López I., Pérez M., Lamuela-Raventós R.M. (2024). Total (poly)phenol analysis by the Folin-Ciocalteu assay as an anti-inflammatory biomarker in biological samples. Crit. Rev. Food Sci. Nutr..

[52] Azizian-Shermeh O., Valizadeh M., Taherizadeh M., Beigomi M. (2019). Phytochemical investigation and synthesis of bioactive nanoparticles using saffron petal extract. Appl. Nanosci..

[53] Ronsisvalle S., Panico A., Santonocito D., Siciliano E.A., Sipala F., Montenegro L., Puglia C. (2023). Evaluation of Crocin Content and In Vitro Antioxidant and Anti-Glycation Activity of Different Saffron Extracts. Plants.

[54] Ali A., Yu L., Kousar S., Khalid W., Maqbool Z., Aziz A., Arshad M.S., Aadil R.M., Trif M., Riaz S. (2022). Crocin: Functional characteristics and food applications. Front. Nutr..

[55] Eghbali S., Farhadi F., Askari V.R. (2023). An overview of analytical methods employed for quality assessment of *Crocus sativus* (saffron). Food Chem. X.

[56] Mottaghipisheh J., Mahmoodi Sourestani M., Kiss T., Horváth Attila Tóth B., Ayanmanesh M., Khamushi A., Csupor D. (2020). Comprehensive chemotaxonomic analysis of saffron crocus tepal and stamen samples, as raw materials with potential antidepressant activity. J. Pharm. Biomed. Anal..

[57] Kabiri M., Rezadoost H., Ghassempour A. (2017). A comparative quality study of saffron constituents through HPLC and HPTLC methods followed by isolation of crocins and picrocrocin. LWT—Food Sci. Technol..

[58] Alkhoury S., Kateeb R., Akasha R., Thallaj N. (2025). Analysis of Crocin Content in Saffron (*Crocus sativus* L) Cultivated in Syria Using Liquid Chromatography- Mass Spectrometry. Am. J. Biomed. Sci. Res..

[59] Zeka K., Ruparelia K.C., Continenza M.A., Stagos D., Vegliò F., Arroo R.R.J. (2015). Petals of *Crocus sativus* L. as a potential source of the antioxidants crocin and kaempferol. Fitoterapia.

[60] Reddy C., Bharate S., Vishwakarma R., Bharate S. (2020). Chemical analysis of saffron by HPLC based crocetin estimation. J. Pharm. Biomed. Anal..

[61] Jafari S.M., Tsimidou M.Z., Rajabi H., Kyriakoudi A. (2020). Chapter 16—Bioactive ingredients of saffron: Extraction, analysis, applications. Saffron: Science, Technology and Health.

[62] Sadeghi S.A., Karami K., Alafchi B., Ranjbar A., Mehri F. (2024). The Concentration of Lead, Cadmium, Copper, Nickel, Zinc, Manganese and Iron in Saffron: A Meta-analysis and Systematic Review. J. Adv. Environ. Health Res..

[63] Askary M., Behdani M.A., Mollaei H., Fallahi H.R. (2023). Evaluation of the Effects of Organic and Conventional Cultivation Practices on Phytochemical and Anti-Cancer Activities of Saffron (*Crocus sativus* L.). J. Agric. Sci. Technol..

[64] Noori P., Hashemi M., Ghasemi S. (2022). A Comprehensive Review of Minerals, Trace Elements, and Heavy Metals in Saffron. Curr. Pharm. Biotechnol..

[65] Kumari L., Tripathy S.S. (2024). Elemental composition and contaminants of saffron from different origins and geographical discrimination using chemometrics. Food Addit. Contam. Part A.

[66] Perini M., Pianezze S., Ziller L., Ferrante M., Ferella F., Nisi S., Foschi M., D’ARchivio A.A. (2020). Stable isotope ratio analysis combined with inductively coupled plasma-mass spectrometry for geographical discrimination between Italian and foreign saffron. J. Mass Spectrom..

[67] D’aRchivio A.A., Di Vacri M.L., Ferrante M., Maggi M.A., Nisi S., Ruggieri F. (2019). Geographical discrimination of saffron (*Crocus sativus* L.) using ICP-MS elemental data and class modeling of PDO Zafferano dell’Aquila produced in Abruzzo (Italy). Food Anal. Methods.

[68] Feizy J., Jahani M., Moradi E., Ahmadi S. (2022). Measurement of minerals in saffron’s stamens, petals and styles. J. Saffron Agron. Technol..

[69] Heydari S., Rezaei R., Haghayegh G.H. (2013). Effect of Drying Processes on Stability of Anthocyanin Extracts from Saffron Petal. Evol. Trends Eng. Technol..

[70] Jabbari N., Goli M., Shahi S. (2024). Optimization of Bioactive Compound Extraction from Saffron Petals Using Ultrasound-Assisted Acidified Ethanol Solvent: Adding Value to Food Waste. Foods.

[71] Foued D., Latifa K., Dems M.A., Allah K.M.S., Fernandez M.J.F., Samira K., Mustapha M.A., Atoki A.V., Saadoun S., Mehdi H. (2025). North African saffron: Chemical characterization, pharmacological properties and toxicity assessment conducted by in silico and in vitro methods. Int. J. Food Prop..

[72] Enaru B., Drețcanu G., Pop T.D., Stǎnilǎ A., Diaconeasa Z. (2021). Anthocyanins: Factors Affecting Their Stability and Degradation. Antioxidants.

[73] Fayssal S.A., Kumar P., Popescu S.M., Khanday M.U.-D., Sardar H., Ahmad R., Gupta D., Gaur S.K., Alharby H.F., Al-Ghamdi A.G. (2024). Health risk assessment of heavy metals in saffron (*Crocus sativus* L.) cultivated in domestic wastewater and lake water irrigated soils. Heliyon.

[74] Ferla G., Mura B., Falasco S., Caputo P., Matarazzo A. (2024). Multi-criteria decision analysis for sustainability assessment in food sector. Sci. Total Environ..

[75] Oprea O.B., Gaceu L. (2016). Application of multiple criteria decision making in bakery industry. Bull. Transilv. Univ. Brașov.

[76] Serpa N.P., da Silva D.J.C., Wegner R.d.S., Stertz E.d.S., Teixeira C.S., Lopes L.F.D. (2022). Quality and sustainability in bakery production using fuzzy decision methods. Environ. Qual. Manag..

[77] Berezina N.A., Artemov A.V., A Nikitin I., Khmeleva E.V., A Kozlova V., A Makarova N. (2021). Environmental assessment of food systems. IOP Conf. Ser. Earth Environ. Sci..

[78] Voccia D., Kum S.W., Suciu N.A., Monaco E., Trevisan M., Lamastra L. (2026). A Multi-Criteria Evaluation Tool for Assessing Circularity in Innovative Bio-Based Solutions from Food Industry By-Products. Appl. Sci..

[79] Fatima G., Khan S., Shukla V., Awaida W., Li D., Gushchina Y.S. (2025). Nutraceutical formulations for management of chronic diseases. Front. Nutr..

[80] Marrone G., Urciuoli S., Di Lauro M., Cornali K., Montalto G., Masci C., Vanni G., Tesauro M., Vignolini P., Noce A. (2024). Saffron and its by-products: Health effects. Nutrients.

[81] Zhang Y., Gong Y., Hu J., Zhang L., Benito M.J., Usmanov D., Nishanbaev S.Z., Song X., Zou L., Wu Y. (2025). Quercetin and kaempferol from saffron petals alleviated hydrogen peroxide-induced oxidative damage in B16 cells. J. Sci. Food Agric..

[82] Frusciante L., Geminiani M., Shabab B., Olmastroni T., Scavello G., Rossi M., Mastroeni P., Nyong’a C.N., Salvini L., Lamponi S. (2024). Exploring the antioxidant and anti-inflammatory potential of saffron (*Crocus sativus*) tepals extract within the circular bioeconomy. Antioxidants.

[83] Yang L., Xu H.H., Hong Q., Xu N., Zhang Y., Tao R., Li S., Zhang Z., Geng J., Wang Z. (2024). *Crocus sativus* L. produces anti-inflammatory effects and regulates the NLRP3–NF-κB pathway. Acupunct. Herb. Med..

[84] Moshfegh F., Balanejad S.Z., Shahrokhabady K., Attaranzadeh A. (2022). *Crocus sativus* (saffron) petals extract and its active ingredient, anthocyanin improves ovarian dysfunction, regulation of inflammatory genes and antioxidant factors in testosterone-induced PCOS mice. J. Ethnopharmacol..

[85] Safizadeh B., Hoshyar R., Hemmati M., Zarban A., Ebrahimi R. (2016). A preliminary evaluation of effects of high doses of Jujube and Saffron on biochemical and hematological parameters in rats. Clin. Phytoscience.

[86] Tabeshpour J., Sobhani F., Sadjadi S.A., Hosseinzadeh H., Mohajeri S.A., Rajabi O., Taherzadeh Z., Eslami S. (2017). A double-blind, randomized, placebo-controlled trial of saffron stigma (*Crocus sativus* L.) in mothers suffering from mild-to-moderate postpartum depression. Phytomedicine.

[87] Kashani L., Eslatmanesh S., Saedi N., Niroomand N., Ebrahimi M., Hosseinian M., Foroughifar T., Salimi S., Akhondzadeh S. (2017). Comparison of saffron versus fluoxetine in treatment of mild to moderate postpartum depression: A double-blind, randomized clinical trial. Pharmacopsychiatry.

